# Regeneration of the pulmonary vascular endothelium after viral pneumonia requires COUP-TF2

**DOI:** 10.1126/sciadv.abc4493

**Published:** 2020-11-25

**Authors:** Gan Zhao, Aaron I. Weiner, Katherine M. Neupauer, Maria Fernanda de Mello Costa, Gargi Palashikar, Stephanie Adams-Tzivelekidis, Nilam S. Mangalmurti, Andrew E. Vaughan

**Affiliations:** 1 Department of Biomedical Sciences, School of Veterinary Medicine, University of Pennsylvania, Philadelphia, PA 19104, USA.; 2 Lung Biology Institute, University of Pennsylvania, Philadelphia, PA 19104, USA.; 3 Department of Clinical Veterinary Medicine, College of Veterinary Medicine, Huazhong Agricultural University, Wuhan 430070, China.; 4 Pulmonary, Allergy, and Critical Care Division, Department of Medicine, Perelman School of Medicine, University of Pennsylvania, Philadelphia, PA 19104, USA.; 5 Institute for Regenerative Medicine, University of Pennsylvania, Philadelphia, PA 19104, USA.

## Abstract

Acute respiratory distress syndrome is associated with a robust inflammatory response that damages the vascular endothelium, impairing gas exchange. While restoration of microcapillaries is critical to avoid mortality, therapeutic targeting of this process requires a greater understanding of endothelial repair mechanisms. Here, we demonstrate that lung endothelium possesses substantial regenerative capacity and lineage tracing reveals that native endothelium is the source of vascular repair after influenza injury. Ablation of chicken ovalbumin upstream promoter–transcription factor 2 (COUP-TF2) (Nr2f2), a transcription factor implicated in developmental angiogenesis, reduced endothelial proliferation, exacerbating viral lung injury in vivo. In vitro, COUP-TF2 regulates proliferation and migration through activation of cyclin D1 and neuropilin 1. Upon influenza injury, nuclear factor κB suppresses COUP-TF2, but surviving endothelial cells ultimately reestablish vascular homeostasis dependent on restoration of COUP-TF2. Therefore, stabilization of COUP-TF2 may represent a therapeutic strategy to enhance recovery from pathogens, including H1N1 influenza and SARS-CoV-2.

## Teaser

Repair of blood vessels after viral lung injury is achieved by resident endothelial cells and requires COUP-TF2.

## INTRODUCTION

The lung frequently encounters injurious toxins and pathogens, necessitating a robust capacity for repair to maintain function. Highly virulent viruses, including pandemic influenza (e.g., 1918 H1N1 “Spanish flu” and H5N1 “bird flu”) and coronavirus strains [severe acute respiratory syndrome coronavirus (SARS-CoV) and SARS-CoV-2], present particularly challenging insults, given that large regions of alveolar epithelium are destroyed during infection (*1*, *2*), resulting in diffuse alveolar damage (DAD) (*3*). While most of these viruses do not directly infect lung endothelium, the robust inflammatory response induced by these pathogens causes significant injury to the pulmonary vasculature. This vascular disruption causes loss of barrier function and pulmonary edema in the complex network of microcapillaries required for gas exchange (*4*, *5*). The resulting acute respiratory distress syndrome (ARDS) is the major cause of mortality in infected individuals (*6*), resulting in as many as 500,000 deaths worldwide annually from influenza alone (*7*) and likely many more this year from SARS-CoV-2 infection [coronavirus disease 2019 (COVID-19)]. Understanding how lung endothelial repair is achieved should facilitate strategies to restore vascular homeostasis and thus circumvent patient mortality.

Despite the central importance of vascular endothelial restoration in the resolution and repair of severe lung injury, viral, or otherwise, little is known as to how this is achieved. Vascular repair in most adult tissues is thought to be achieved by reentry of preexisting endothelial cells (ECs) into the cell cycle, i.e., angiogenic proliferation. This has been definitively demonstrated in the heart and aorta (*8*, *9*). However, reports of circulating, bone marrow–derived cells being recruited to injured vascular beds and transdifferentiating into nascent ECs have challenged this paradigm (*10*–*13*), leading to confusion as to the most relevant source of endothelial repair. Attempts to therapeutically enhance endothelial repair in the lung, and thereby prevent or blunt the severity of viral pneumonia, require a cohesive understanding of both the cells of origin and molecular drivers of endothelial reconstitution.

Our studies here lay the groundwork for the development of a comprehensive model for pulmonary vascular endothelial regeneration. We directly demonstrate significant proliferation of the lung endothelium after influenza injury and demonstrate that blood vessel restoration is derived entirely from preexisting ECs with no evidence for vasculogenic contribution from other cell lineages. We implicate the orphan hormone receptor/transcription factor COUP-TF2 (chicken ovalbumin upstream promoter–transcription factor 2) as a central regulator of this process, responsible for driving EC proliferation and migration through direct transcriptional activation of the cell cycle regulator CCND1 (cyclin D1) and by enhancing VEGFA/VEGFR2 (vascular endothelial growth factor A/vascular endothelial growth factor receptor 2) signaling through activation of NRP1 (neuropilin 1). We also demonstrate that proinflammatory cytokine activators of nuclear factor κB (NF-κB) repress COUP-TF2 early after influenza infection, potentially inhibiting an earlier and more robust regenerative response. This work implicates COUP-TF2 as a master regulator and potential therapeutic target for enhancing pulmonary vascular endothelial repair.

## RESULTS

### Endothelial regeneration during influenza-induced lung injury

**Fig. 1:**
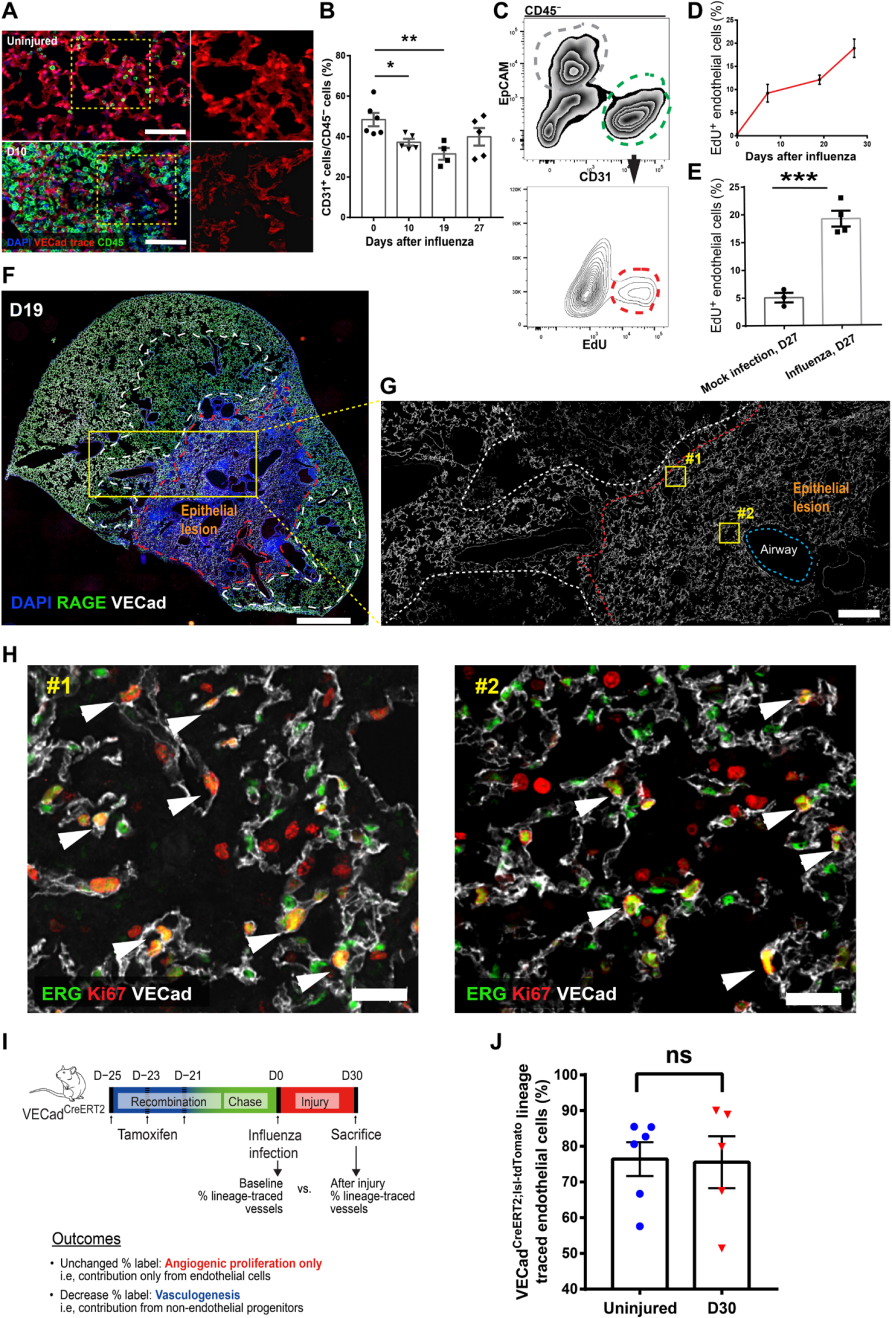
Injury and proliferation of ECs after influenza infection in vivo. (**A**) Representative immunofluorescence images of capillaries (red) and leukocytes (green) in lung tissue with or without influenza injury. Scale bars, 50 μm. D10, day 10. (**B**) Quantification of ECs (CD31^+^) in the nonleukocyte fraction (exclusion of CD45^+^ cells) via flow cytometric analysis. (**C**) Representative gating scheme for identification of proliferating EC analysis via EdU incorporation at day 19 after influenza. (**D**) Intracellular flow cytometry quantification of proliferative ECs (CD45^−^/EpCAM^−^/CD31^+^/EdU^+^) as a percentage of total lung ECs on days 0, 7, 19, and 27 after influenza infection. *n* = 3 to 4 per group. (**E**) Cumulative endothelial proliferation on day 27 after infection and mock-infected controls. (**F**) Typical image of lungs at day 19 after influenza. Red dashes represent the lung epithelial lesion area (RAGE^−^), and the white dashes area represents the VECad low-expression area. Scale bar, 1 mm. (**G**) The enlarged area from (F) shows the vascular endothelium across normal and injured epithelial areas (white, VECad). Scale bar, 200 μm. (**H**) Representative immunostaining of proliferative ECs from peripheral (#1) and central (#2) parts of epithelial lesion on day 19. Arrows indicate proliferative ECs (colocalization of ERG and Ki67). Scale bars, 25 μm. (**I**) Methodology to determine whether regenerated ECs are derived from preexisting endothelium or transdifferentiation of another cell type. (**J**) Statistical analysis of the level of lineage-traced ECs in uninjured and day 30 after infection mice. Each point in (B), (E), and (J) represents one mouse. Data in (B) were calculated using one-way analysis of variance (ANOVA), followed by Dunnett’s multiple comparison test; data in (E) and (J) were calculated using unpaired two-tailed *t* test. Data are presented as means ± SEM. *P < 0.05, **P < 0.01, and ***P < 0.001. ns, not significant.

Before addressing the mechanisms facilitating endothelial regeneration after viral injury, we sought to better characterize influenza-induced vascular disruption. We initially infected VECad^CreERT2;lsl-tdTomato^ mice with influenza A strain PR8, which causes heterogeneous damage similar to that described for human infection (*14*–*16*) and allows for specific identification of ECs by tdTomato expression (*17*). As shown in Fig. 1A, the capillary plexus surrounding the alveoli was obviously disturbed, as evidenced by profound CD45^+^ leukocyte infiltration and marked disruption of the vasculature network by day 10 after influenza infection. We quantified the number of ECs after influenza injury using flow cytometry, observing a significant decrease in ECs on days 10 and 19 after injury before trending toward restoration by day 27, although ECs still did not completely return to baseline levels (Fig. 1B). Nonetheless, this suggested that the pulmonary endothelium has a capacity for regeneration after injury. To confirm this, we administered the nucleoside analog 5-ethynyl-2-deoxyuridine (EdU) (50 mg/kg, intraperitoneally) three times weekly throughout the entire course of injury (27 days), allowing for unambiguous quantification of cells that proliferated at any point during injury and repair. Meanwhile, uninfected mice [mock infected with phosphate-buffered saline (PBS)] were administered the same dose of EdU over the same time course to assess homeostatic levels of proliferation. Intracellular EdU flow analysis was used to quantify proliferative ECs after excluding epithelial and immune cells. ECs exhibited demonstrable but low levels of EdU incorporation (CD45^−^/EpCAM^−^/CD31^+^/EdU^+^) under uninjured conditions, but by day 27, ~20% were EdU^+^ (Fig. 1, C to D), indicating that, at minimum, 15% of the ECs were newly generated after influenza injury (Fig. 1E).

Using in situ immunofluorescence analysis, we unambiguously identified proliferating ECs based on nuclear colocalization of Ki67 with ERG (ETS-related gene), a highly specific transcription factor expressed in the endothelium (*18*). Notably, scattered endothelial proliferation was observed after 12 days of influenza injury (fig. S1, A and B), which was significantly increased around day 19 (Fig. 1 F to H), while very few proliferating ECs were observed by day 27 (fig. S1, D and E), indicating that the endothelial repair process was essentially accomplished. Using loss of the alveolar epithelial marker RAGE (receptor for advanced glycation endproducts) to distinguish influenza-injured areas, we found that proliferating ECs were mainly distributed in the damaged area of epithelium (Fig. 1, F to H), consistent with the model that endothelial injury is secondary to the primary epithelial insult. Curiously, at days 12 and 19, we observed some regions with reduced staining for the endothelial maker VECad, which were adjacent to but distinct from the epithelial lesions and largely lacked proliferating ECs. However, this phenomenon ceased by day 27, suggesting that some ECs may undergo some sort of transient “adaptive transformation” during influenza injury. Together, this indicates that endothelial regeneration occurs after influenza infection.

### Lineage trace validation of angiogenic proliferation from preexisting endothelium

Previous studies in the lung and other tissues have suggested that circulating, bone marrow–derived progenitor cells are involved in endothelial repair (i.e., vasculogenesis), but these studies remain controversial. To determine whether regenerated ECs after influenza injury are exclusively derived from proliferation of preexisting endothelium or whether vasculogenesis may also be involved, VECad^CreERT2;lsl-tdTomato^ mice were subject to PR8 infection and euthanized 30 days later when mouse weights have fully recovered (fig. S2A). In these mice, any cells that acquire endothelial identity during or after injury will not express tdTomato. Thus, if non-ECs contribute to endothelial regeneration (vasculogenesis), then the fraction of lineage-labeled cells should decrease after injury, whereas there will be no change if preexisting ECs alone are responsible for repair (Fig. 1I). Immunostaining for endothelial markers CD31 and vascular endothelial cadherin (VECad) showed that essentially all recovered ECs still bore the lineage label (fig. S2B). Highly quantitative flow cytometry confirmed that the fraction of labeled cells after injury was not significantly altered between baseline levels and day 30 after infection (Fig. 1J and fig. S2C). Together, these results suggest that angiogenic proliferation of surviving ECs is the main mechanism of endothelial repair after viral lung injury.

### Endothelial suppression of COUP-TF2 by influenza injury

**Fig. 2:**
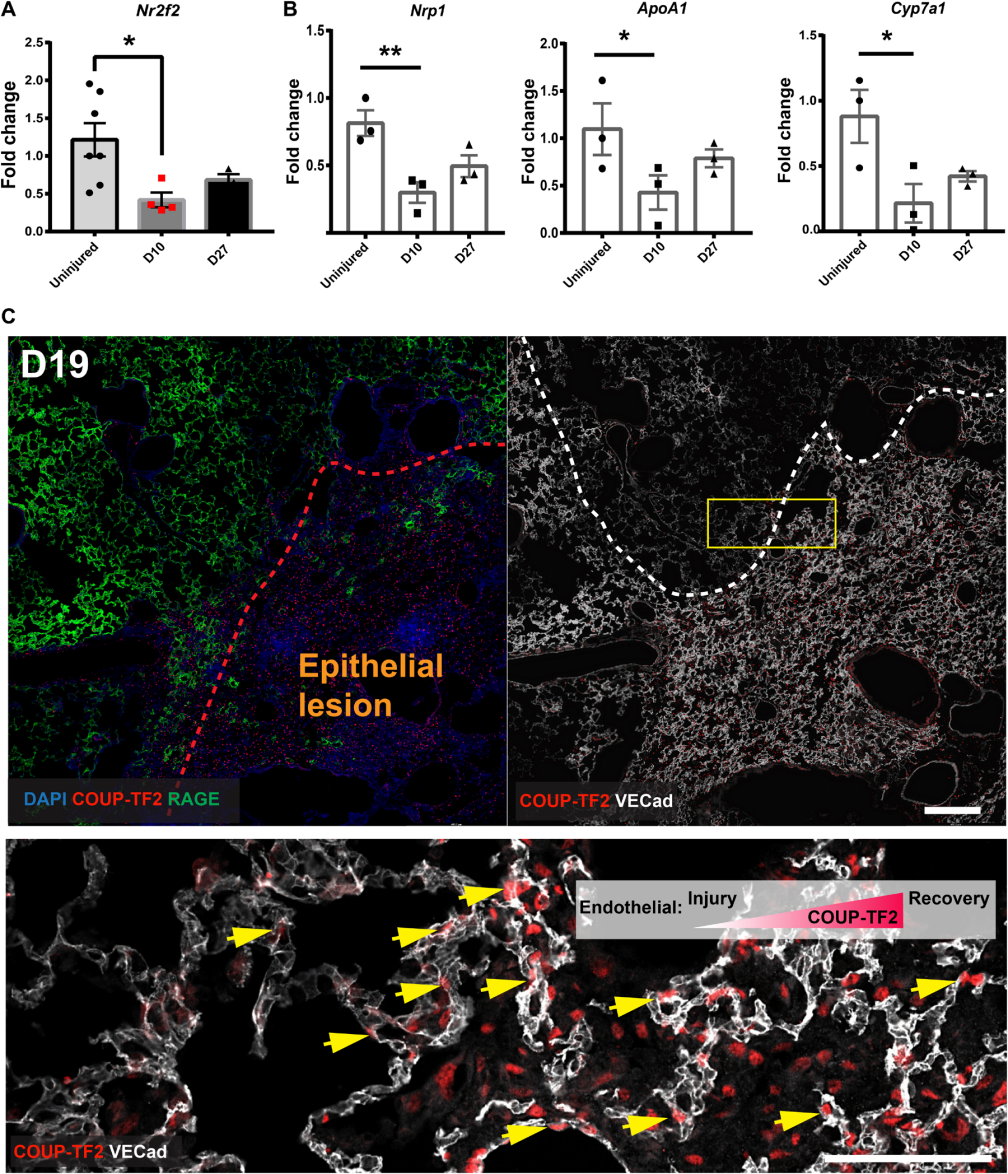
COUP-TF2 is down-regulated in ECs upon influenza injury. (**A**) qPCR analysis of COUP-TF2 in isolated lung ECs (CD45^−^CD31^+^) sorted on days 0 (uninjured), 10, and 27. (**B**) qPCR analysis of COUP-TF2 target genes in lung ECs sorted as in (A). Each dot represents a single mouse. (**C**) Immunofluorescence staining showing COUP-TF2 expression level in the capillaries in normal and injured epithelial regions on day 19 after influenza. The red dashes represent the lung epithelial lesion area (RAGE^−^), and the white dashes represent the VECad low-expression area. Scale bars, 200 μm (top) and 100 μm (bottom). Arrows indicate endothelial COUP-TF2 expression in capillaries. Data are presented as means ± SEM. *P < 0.05 and **P < 0.01 by one-way ANOVA, followed by Dunnett’s multiple comparison test.

Having demonstrated that pulmonary endothelial regeneration does occur and is mediated by native ECs themselves, we turned our focus to identification of molecular drivers of this regenerative phenomenon. COUP-TF2, an orphan hormone receptor and transcription factor, has been previously implicated in EC fate choice (*19*), angiogenesis, and coronary artery development (*20*), and COUP-TF2 is expressed in all lung EC types other than arteries in mice and humans (fig. S3). To determine whether COUP-TF2 is involved in the repair of influenza-mediated vascular injury, we isolated ECs (CD45^−^/CD31^+^) from mice on days 0 (uninjured), 10, and 27 after infection by fluorescence-activated cell sorting (FACS). Quantitative polymerase chain reaction (qPCR) analysis for COUP-TF2 mRNA showed significant down-regulation in lung ECs after influenza injury, which slowly recovered concurrent with endothelial proliferation (Fig. 2A). We also observed down-regulation and slow recovery of three COUP-TF2 target genes apolipoprotein A1 (*ApoA1*) (*21*), *Nrp1* (*22*), and cytochrome P450, family 7, subfamily a, polypeptide 1 (*Cyp7a1*) (*23*) in ECs (Fig. 2B), consistent with the expression trend of COUP-TF2 itself. With regard to the spatiotemporal expression pattern of COUP-TF2 during endothelial repair, immunostaining indicates that the expression of COUP-TF2 was reduced on days 12 and 19 after influenza infection and large regions lacking appreciable COUP-TF2 were present (Fig. 2C and fig. S1C). On day 19, the expression of COUP-TF2 in regions bearing proliferative ECs (RAGE^−^ epithelial lesions) was strongly increased (Fig. 2C). By day 27, with the endothelial repair basically completed, COUP-TF2 expression was essentially normalized across the tissue (fig. S1F). Given these dynamics of COUP-TF2 and COUP-TF2 targets, we assessed a potential functional role for COUP-TF2 in lung endothelial repair.

### Endothelial ablation of COUP-TF2 exacerbates influenza-induced lung injury

**Fig. 3:**
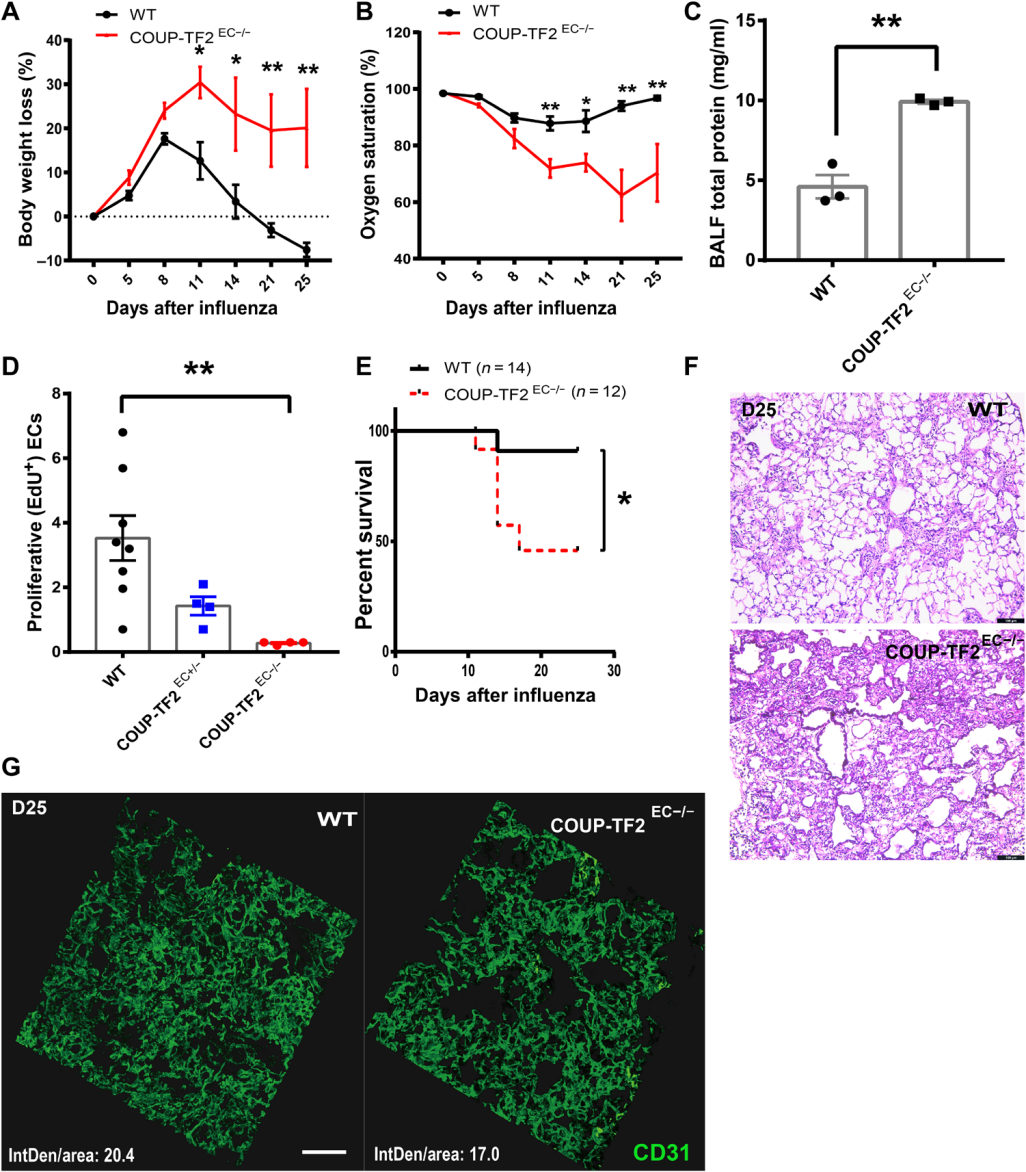
Endothelial COUP-TF2 deletion in vivo prevents endothelial repair after influenza injury. VECad^CreERT2^;COUP-TF2^flox/flox^ or WT (VECad^CreERT2^ or COUP-TF2^flox/flox^) mice were administered five doses of tamoxifen, followed by 1 week of chase and influenza infection. Time course of changes in capillary oxygen saturation (**A**) and weight loss (**B**) was observed in WT, COUP-TF2^EC+/−^, and COUP-TF2^EC−/−^ mice. *n* = 3 to 5 per group. (**C**) BALF was collected on the day 11 after infection for each group of mice, and the concentration of total protein in lavage fluid was detected by bicinchoninic acid (BCA) protein assay. (**D**) Endothelial EdU incorporation was measured by flow cytometry in lungs from WT and COUP-TF2^EC−/−^ mice (as in Fig. 1, D and E). (**E**) Kaplan-Meier survival curves after influenza infection. (**F**) Histological changes in the lungs of WT and COUP-TF2^EC−/−^ influenza-challenged mice at day 25 after infection. Scale bars, 100 μm. (**G**) Lungs were harvested on day 25 after infection, fixed, and sliced into 50-μm sections. Slices were mounted, stained for CD31, and imaged on a confocal microscope. Scale bar, 50 μm. IntDen indicates integrated density of CD31 fluorescence. Each point represents one mouse. Data in (A) to (D) were calculated using unpaired two-tailed *t* test; data in (E) were calculated using log-rank test. Data are presented as means ± SEM. *P < 0.05 and **P < 0.01.

To study the physiological impact of COUP-TF2 in pulmonary endothelial repair, we crossed VECad^CreERT2^ mice with COUP-TF2^flox^ mice (*24*) to generate both heterozygous and homozygous mutant mice and then proceeded with COUP-TF2 deletion in adult mice via tamoxifen administration (fig. S4A). Unchallenged mice did not exhibit any overt phenotypes 2 months after COUP-TF2 deletion (fig. S4, B to E). Upon influenza infection, however, COUP-TF2^EC−/−^ (VECad^CreERT2^;COUP-TF2^flox/flox^) mice became moribund by day 11 after infection, exhibiting exaggerated weight loss, strongly decreased capillary oxygen saturation, and increased total protein in the bronchoalveolar lavage fluid (BALF), and surviving COUP-TF2^EC−/−^ mice remained ill and did not appreciably recover body weight or blood oxygenation levels through 25 days after infection (Fig. 3, A to C). To assess whether EC proliferation was affected by COUP-TF2 loss, we administered a single dose of EdU to mice 9 days after infection and isolated lung ECs at 10 days after infection. COUP-TF2–deficient ECs exhibited markedly decreased proliferation (EdU incorporation) (Fig. 3D and fig. S5), indicating that a lack of angiogenic proliferative capacity is a major contributor to the increased pathology. The survival curve analysis showed that around 90% of control mice (mice having only VECad^CreERT2^ or COUP-TF2^flox/flox^ mice lacking CreERT2) were able to survive indefinitely (up to 25 days after infection), while ablation of COUP-TF2 (COUP-TF2^EC−/−^) significantly reduced the survival to less than 50% (Fig. 3E). Histological analysis of the COUP-TF2^EC−/−^ lungs in the mice that did survive to day 25 clearly demonstrated more severe injury, inflammation, and remodeling, as assessed by the destruction of alveolar architecture, leukocyte infiltration, alveolar interstitial thickening, and apparent fibrosis (Fig. 3F). Moreover, confocal analysis showed that COUP-TF2^EC−/−^ lungs exhibit more severe disruption of intervascular connectivity, increased abnormal vessel space, and lower vascular density compared to the wild-type (WT) group (Fig. 3G). These results indicate a critical requirement for COUP-TF2 in effective vascular repair and regeneration in vivo.

### Proinflammatory cytokines suppress COUP-TF2 expression in vitro

**Fig. 4:**
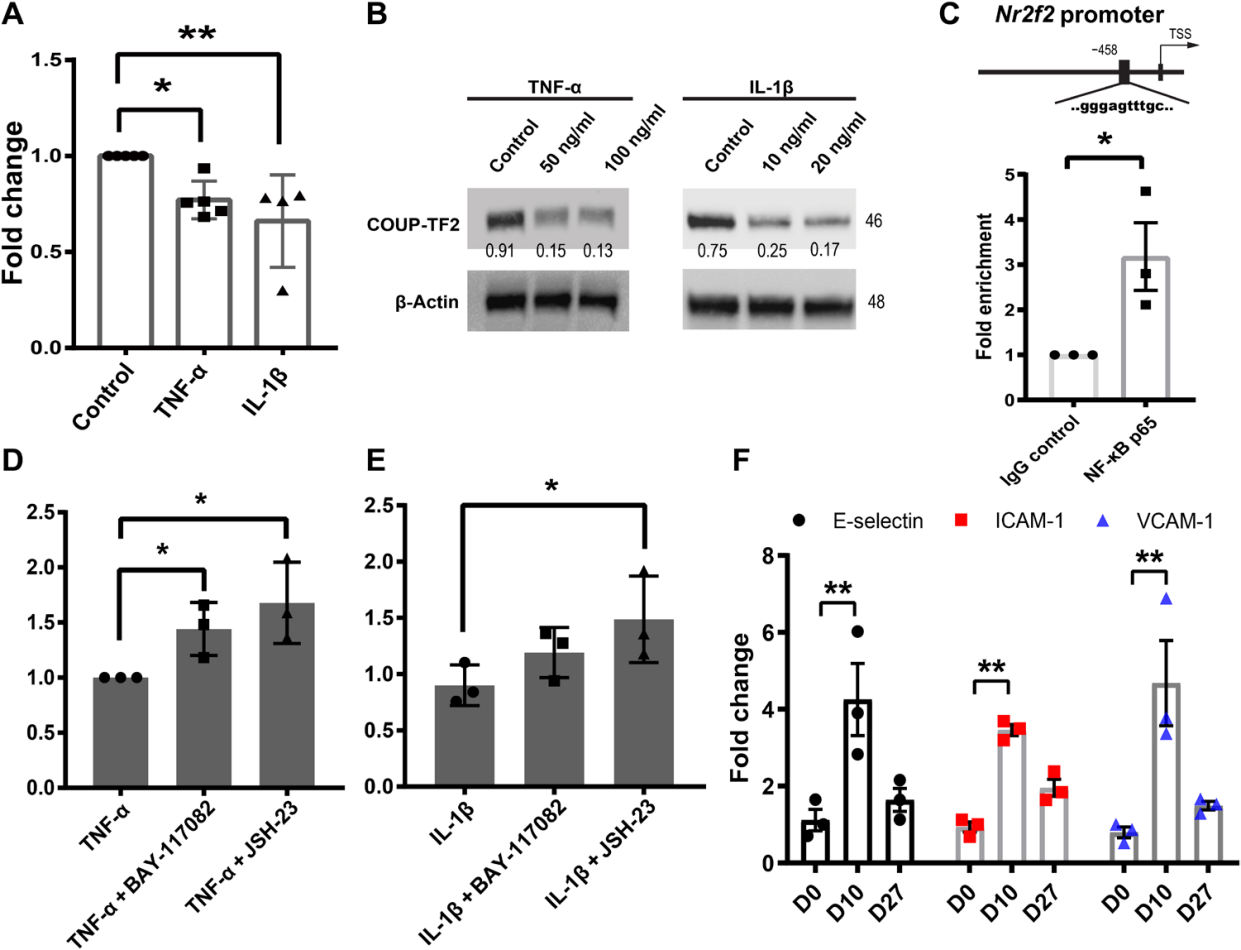
COUP-TF2 is inhibited by TNF-α– and IL-1β–mediated NF-κB activation in vitro. (**A**) qPCR analysis of COUP-TF2 expression in iMVECs treated with either TNF-α or IL-1β. (**B**) Western blot analysis of COUP-TF2 expression in iMVECs treated with either TNF-α or IL-1β. Numbers under Western blot bands represent relative quantifications over actin. *n* = 3. (**C**) ChIP-qPCR was performed to amplify the target region in the *COUP-TF2/Nr2f2* promoter. iMVECs were pretreated for 2 hours with NF-κB inhibitors BAY-117082 (5 μM) and JSH-23 (5 μM), followed by addition of either TNF-α or IL-1β for 24 hours. qPCR analysis of COUP-TF2 in both TNF-α (**D**) and IL-1β (**E**) treatment group. (**F**) qPCR analysis of NF-κB target genes E-selectin, ICAM-1, and VCAM-1 in isolated lung ECs (CD45^−^CD31^+^) sorted on days 0 (uninjured), 10, and 27. Each dot represents one independent experiment or one mouse. Data in (A), (D), and (E) are presented as means ± SD. Data in (C) and (F) are presented as means ± SEM. Data in (A), (C), (D), and (E) were calculated using unpaired two-tailed *t* test; data in (F) were calculated using two-way ANOVA, followed by Dunnett’s multiple comparison test. *P < 0.05 and **P < 0.01. TSS, transcriptional start site.

Despite the requirement for COUP-TF2 in lung endothelial repair, its expression levels were, seemingly paradoxically, decreased early after infection (Fig. 2A). Proinflammatory cytokines such as interleukin-1β (IL-1β) and tumor necrosis factor–α (TNF-α) are both strongly induced shortly after PR8 influenza infection in mice (*25*–*27*), and, notably, COUP-TF2 expression is suppressed by these same cytokines in endometrial stromal cells (*28*). To determine whether these cytokines are similarly responsible for endothelial suppression of COUP-TF2 during influenza infection, we treated immortalized human lung microvascular ECs (iMVECs) (*29*) with either IL-1β or TNF-α for 24 hours and confirmed that both cytokines down-regulate COUP-TF2 in iMVECs at both the mRNA and protein level (Fig. 4, A and B). TNF-α and IL-1β are both potent activators of canonical NF-κB, which is a master regulator of inflammatory processes in a wide variety of scenarios, and activation of the NF-κB pathway has been observed in influenza virus–infected mice (*30*). We therefore hypothesized that these cytokines inhibit COUP-TF2 via activation of NF-κB. First, binding sites for NF-κB p65 in the COUP-TF2/*Nr2f2* promoter region were identified, and p65 enrichment was confirmed by chromatin immunoprecipitation (ChIP)–qPCR (Fig. 4C). Next, iMVECs were pretreated with the canonical NF-κB pathway inhibitors, BAY 11-7082 (*31*) (5 μM) and JSH-23 (*32*) (5 μM) for 2 hours before the addition of TNF-α or IL-1β for 24 hours. As predicted, qPCR analysis showed that COUP-TF2/*Nr2f2* was rescued by both inhibitors, although to a lesser degree with BAY 11-7082 in IL-1β treatment (Fig. 4, D and E). Last, well-validated NF-κB target genes intercellular adhesion molecule–1 (ICAM-1), vascular cell adhesion molecule–1 (VCAM-1), and E-selectin (*33*) were assessed in mouse ECs sorted from days 0, 10, and 27, all of which exhibited an inverse expression pattern to that of COUP-TF2, further supporting the conclusion that NF-κB activity inhibits COUP-TF2 (Fig. 4F). These results indicate that proinflammatory cytokines IL-1β and TNF-α can inhibit the expression of COUP-TF2 in ECs by activating NF-κB. Thus, carefully timed inhibition of NF-κB may preserve COUP-TF2 levels and enhance endothelial repair in vivo.

### COUP-TF2 is required for angiogenic proliferation and migration in vitro

**Fig. 5:**
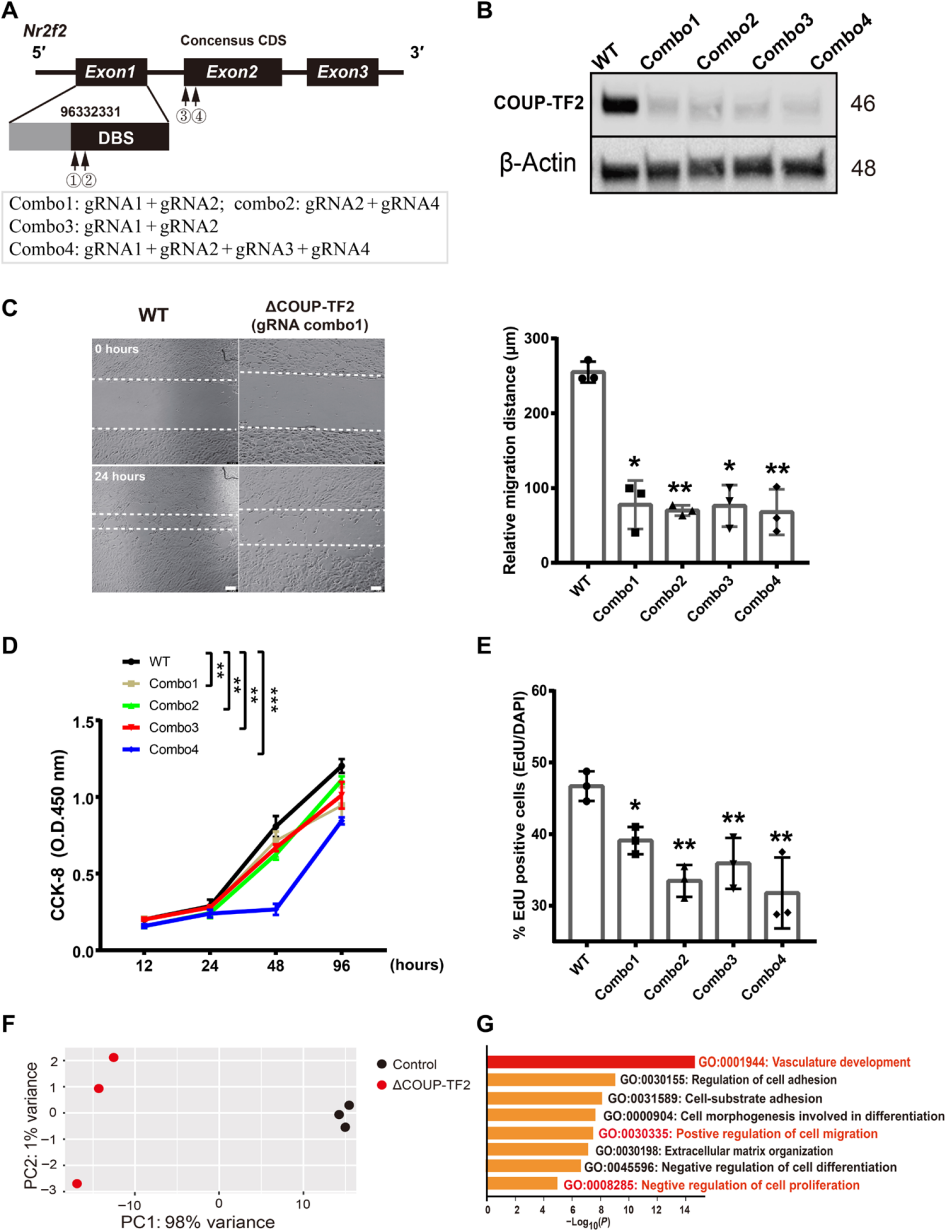
COUP-TF2 deletion inhibits migration and proliferation in vitro. (**A**) Schematic representation of the genomic DNA sequence of COUP-TF2/Nr2f2 with exons numbered. Arrows indicate the sequence targeted by gRNA in CRISPR-Cas9 system; four gRNA combos were designed as shown in the inset box below. DBS, DNA Binding Site. (**B**) Western blot analysis of COUP-TF2 protein expression in iMVECs to confirm KO efficiency, shown as representative blots. *n* = 3. (**C**) Cell migration as assessed by wound or scratch assays with control and COUP-TF2–deleted iMVECs. Photos were taken at the start of the experiment (0 hours) and 24 hours later (24 hours). Images of representative experiments are shown and quantified on the right plot. Scale bars, 100 μm. The effects of COUP-TF2 deletion on cell proliferation were assessed by CCK-8 assay (**D**) and EdU incorporation staining (**E**). O.D., optical density. RNA-seq of COUP-TF2–deleted iMVECs, (**F**) principal components analysis (PCA) representing transcriptomic changes upon COUP-TF2 deletion, demonstrating COUP-TF2 directly or indirectly regulates many target genes in ECs. (**G**) Selected Gene Ontology (GO) pathways of genes down-regulated in COUP-TF2–deficient iMVECs, revealing significantly modulated functional pathways. Angiogenic-related processes are highlighted in red. Each dot represents one independent experiment. Data in (C) and (E) were calculated using one-way ANOVA, followed by Dunnett’s multiple comparison test; data in (D) were calculated using two-way ANOVA, followed by Dunnett’s multiple comparison test. Data are presented as means ± SD. *P < 0.05 and **P < 0.01.

To better address the mechanism by which COUP-TF2 mediates endothelial regeneration in vivo, we turned again to in vitro experiments using iMVECs. Lentivirus encoding Cas9 and multiple guide RNAs (gRNAs) (LentiCRISPRv2) directed toward key domains in the COUP-TF2 gene was generated and used to transduce iMVECs (Fig. 5A), resulting in nearly complete loss of COUP-TF2 protein (Fig. 5B). Since angiogenic repair is thought to involve cell migration, we used a scratch assay to demonstrate that COUP-TF2 knockout (KO) (ΔCOUP-TF2) cells exhibited impaired migration (Fig. 5C). Moreover, ΔCOUP-TF2 cells exhibited significantly lower proliferation as measured by EdU incorporation and a colorimetric proliferation assay CCK-8 (cell counting kit-8) (Fig. 5, D and E, and fig. S6) (*34*). To control for any effects of immortalization in iMVECs, we further validated the proproliferative and promigratory functions of COUP-TF2 in primary human lung microvascular ECs. Expression of COUP-TF2 was significantly inhibited by transfection with a specific *Nr2f2* small interfering RNA (siRNA) (si-Nr2f2) (fig. S7, A and B), and COUP-TF2 knockdown in primary ECs similarly inhibited migration ability and cell proliferation potential (fig. S7, C and D).

RNA sequencing (RNA-seq) of ΔCOUP-TF2 iMVECs was used to further examine the consequences of COUP-TF2 loss during endothelial repair. Principal components analysis (PCA) analysis was performed, with ΔCOUP-TF2 samples forming a tight cluster distinct from WT cells, confirming that COUP-TF2 deletion results in significant transcriptional variation (Fig. 5F). We next analyzed the ~120 differentially expressed genes between these groups to identify candidate transcriptional modules driven by COUP-TF2. Pathways and process enrichment analysis were carried out by Metascape (*35*), demonstrating significant enrichment in blood vasculature development, regulation of cell migration, cell proliferation, and adhesion (Fig. 5G).

We also asked whether overexpression of COUP-TF2 by lentiviral transduction (fig. S8A) might further enhance COUP-TF2–mediated migration and proliferation. The proliferation and migration rate of COUP-TF2–overexpressing (COUP-TF2-OE) cells were markedly increased compared to that of control cells (fig. S8, B to D), implicating COUP-TF2 as a major driver of key components of endothelial regeneration: cell proliferation and migration.

### COUP-TF2 mediates angiogenic repair through direct promoter binding and activation of CCND1 and NRP1

**Fig. 6:**
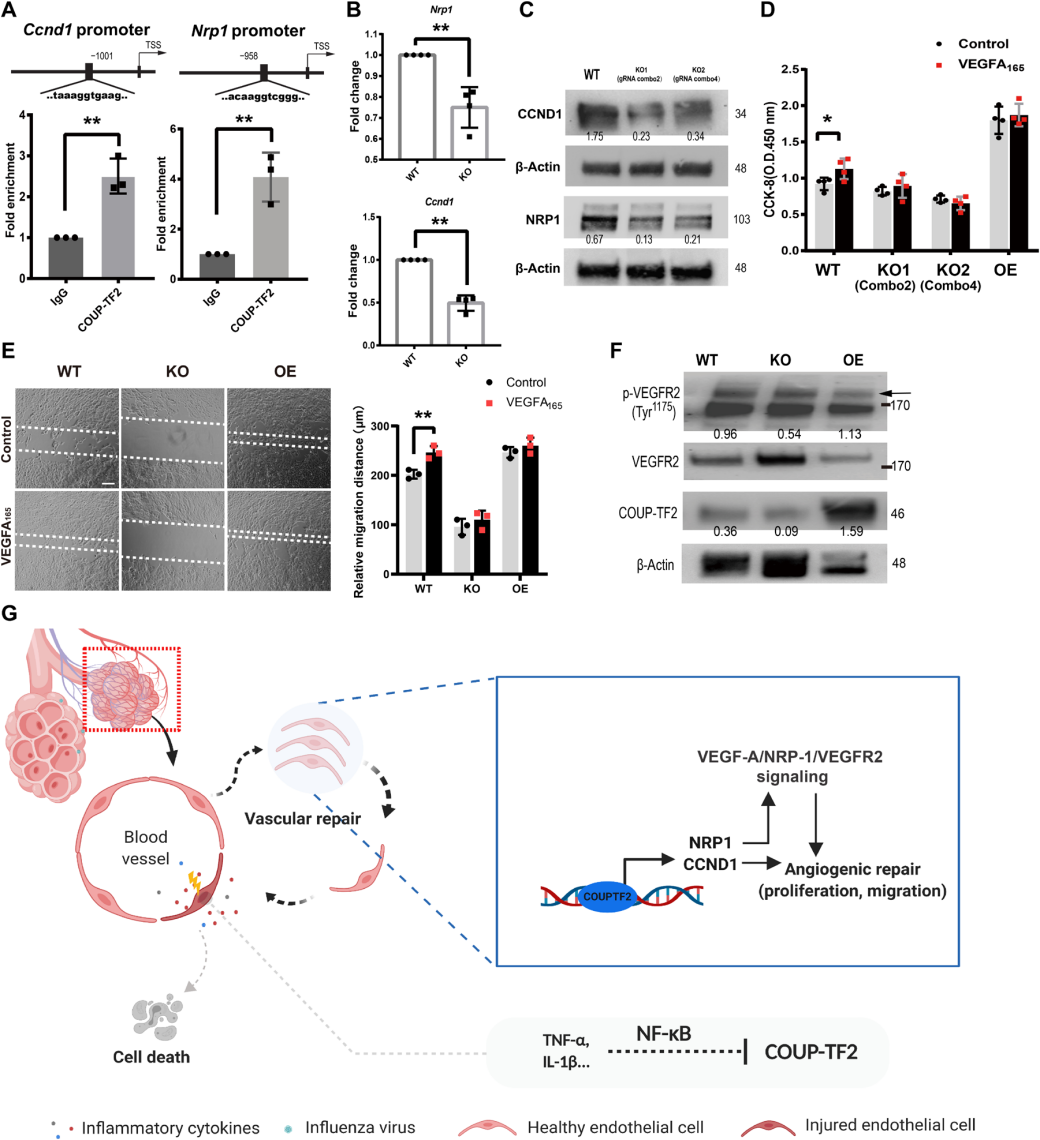
COUP-TF2 directly targets CCND1 and NRP1 to mediate cell proliferation and migration. (**A**) ChIP followed by qPCR was performed to amplify the target region in the *Ccnd1* and *Nrp1* promoters. (**B**) qPCR analysis of COUP-TF2 in WT and COUP-TF2 KO iMVECs. (**C**) Immunoblotting of CCND1 and NRP1 in both WT and KO iMVECs, numbers under Western blot bands represent relative quantifications over actin. *n* = 2. (**D**) CCK-8 assay showing cell proliferation of iMVECs treated ±VEGFA_165_ (20 ng/ml) for 72 hours. *n* = 4. (**E**) Cell migration was assessed using a wound scratch assay. Images were obtained at (0 hours) and (24 hours). Representative photos illustrate scratch closing, quantified in the graph (right). Scale bar, 100 μm. (**F**) Immunoblotting analysis of indicated proteins in WT, COUP-TF2-KO, and COUP-TF2-OE iMVECs treated ±VEGFA_165_ (20 ng/ml) for 30 min, demonstrating VEGFR phosphorylation dependent on COUP-TF2 levels. Numbers under Western blot bands represent relative quantifications over actin. *n* = 2. (**G**) Graphical abstract. Each dot represents one independent experiment. Data are presented as means ± SD. *P < 0.05 and **P < 0.01, calculated by unpaired two-tailed *t* test.

COUP-TF2 can function as either a transcriptional activator or repressor through direct binding to target gene promoters in diverse cellular contexts (*36*), and previous studies indicate that COUP-TF2 can serve as a cell cycle regulator to modulate endothelial proliferation (*19*, *22*). *Ccnd1*, encoding the growth regulator cyclin D1 that functions during developmental angiogenesis (*37*), is ~3.2-fold down-regulated in ΔCOUP-TF2 via RNA-seq, representing a likely critical target gene of COUP-TF2 during endothelial proliferation. Another COUP-TF2 target gene, *Nrp1* (*38*), acts a co-receptor for the VEGFA_165_ isoform of VEGFA, is essential for normal angiogenesis (*39*, *40*), and is significantly reduced in lung ECs after influenza injury (Fig. 2B). We therefore examined whether either of these genes represent direct downstream targets of COUP-TF2. We identified COUP-TF2 binding sites in the *Nrp1* and *Ccnd1* promoters using the COUP-TF2–predicted motif from the JASPAR database and designed primers that surrounded these sequences. ChIP-qPCR revealed that COUP-TF2 binds the promoters of both *Nrp1* and *Ccnd1* (Fig. 6A). Moreover, both *Nrp1* and *Ccnd1* were down-regulated in COUP-TF2 KO iMVECs (Fig. 6B) by qPCR analysis. Immunoblotting of iMVECs confirmed marked loss of CCND1 and NRP1 protein expression in the COUP-TF2 KO cells (Fig. 6C). This demonstrates that COUP-TF2 directly binds and activates the transcription of *Nrp1* and *Ccnd1*.

Nrp1 can enhance VEGFA-driven signaling through VEGFR2 (*40*). VEGFR2 forms a complex with NRP1 after VEGFA_165_ binding, leading to VEGFR2 phosphorylation and activation of the signaling pathway involved in cell migration and angiogenic sprout formation (*40*, *41*). To investigate whether the deletion of COUP-TF2 attenuated the angiogenic effect mediated by VEGFA/VEGFR2 signaling, we evaluated the dependency on COUP-TF2 for VEGFA_165_-induced cell proliferation and migration. iMVECs were incubated for 24, 48, and 72 hours with or without VEGFA_165_, and proliferation was assessed. As predicted, COUP-TF2 deletion slightly but significantly attenuated the mitotic effects of VEGFA_165_ on iMVECs (Fig. 6D and fig. S9A). Moreover, VEGFA_165_ more clearly induced the migration of iMVECs, which was abolished by COUP-TF2 deletion (Fig. 6E). As shown earlier (fig. S8), overexpression of COUP-TF2 enhanced proliferation and migration but was unable to be further boosted by VEGFA_165_ treatment (Fig. 6, D and E), likely due to direct up-regulation of CCND1 or cell cycle activation overwhelming any additional mitogenic effects from NRP1/VEGFR2 signaling. Blocking VEGFA_165_ binding to NRP1 using a selective NRP1 inhibitor, ATWLPPR Peptide TFA (trifluoroacetic acid) (*42*), inhibited migration, although this effect did not reach statistical significance and NRP1 inhibition did not significantly affect VEGFA_165_-induced proliferation (fig. S9, B and C). These results suggest that COUP-TF2–mediated proliferation largely relies on CCND1, whereas NRP1 may have more impact on cell migration. We then asked whether VEGFR2-dependent signaling was affected by COUP-TF2. iMVECs were treated with VEGFA_165_ for 30 min and then collected to examine the VEGFR2 phosphorylation via Western blotting. As shown in Fig. 6F, deletion of COUP-TF2 decreased the phosphorylation of VEGFR2 at Tyr^1175^, while COUP-TF2 overexpression slightly enhanced the phosphorylation of VEGFR2. In summary, COUP-TF2 promotes the transcription of NRP1, which acts to amplify VEGFA_165_-induced angiogenesis: After binding to VEGFA_165_, NRP1 forms a complex with VEGFR2 causing VEGFR2 phosphorylation at Tyr^1175^, thereby activating downstream angiogenic signaling pathways and promoting endothelial migration and proliferation. Loss of COUP-TF2 leads to a decrease in NRP1 expression, thereby inhibiting NRP1/VEGFR2-mediated angiogenesis (fig. S9D).

## DISCUSSION

ECs account for approximately 30% of the total cells in the lung (*43*), lining the capillary network around the alveoli and providing a semipermeable barrier essential for gas exchange. Pulmonary ECs play a critical role in the pathogenesis of viral lung injuries including H1N1 influenza and SARS-CoV-2 (*4*, *44*–*46*) not only by continuing to facilitate gas exchange but also by regulating leukocyte trafficking, influencing coagulation, providing a fluid barrier, and preventing viral dissemination into circulation (viremia). The cause of EC injury in viral pneumonia is thought to be excessive exposure to proinflammatory cytokines secreted by epithelial cells, leukocytes, and macrophages (*4*). Direct endothelial infection with some viruses can also occur (e.g., avian influenza H5N1), which can lead to prolonged endothelial activation, increased permeability, cell death, and vascular destruction, further disrupting the epithelial-endothelial barrier (*4*, *47*). In this study, we used H1N1 influenza A strain PR8 to induce viral pneumonia-like disease in mice, which causes heterogeneous injury similar to so-called DAD that is described for human lung injury (*14*, *16*, *48*). Our data confirm that the pulmonary vascular endothelium is severely damaged during influenza infection, manifested by a reduced proportion of ECs and the obvious disruption of vascular networks. We also observed that the fraction of vascular ECs was still lower than normal even after mice seemingly recovered from infectious symptoms, indicating the possibility of permanent injury or long-term sequelae and likely persistent chronic inflammation as observed in other recent studies (*48*, *49*).

While incomplete, overt recovery does occur as judged by at least partial restoration of pulmonary function and body weight, prompting our hypothesis that the pulmonary endothelium has a capacity for regeneration and/or restoration of homeostatic function after injury. Our data clearly evidenced that around 15% of ECs by 27 days after influenza infection are newly generated (incorporated EdU) and lineage tracing confirmed that essentially all these regenerated cells were derived from preexisting, resident endothelium, i.e., “angiogenic” proliferation, which is consistent with recent studies in other tissues (*8*, *9*). Because of the inherent difficulty imaging the pulmonary capillary plexus, it remains uncertain whether lung endothelial repair occurs via sprouting and elongation of new vessels from existing vessels (sprouting angiogenesis) or by remodeling of existing vessels via internal division of preexisting vessels in the capillary plexus (intussusceptive angiogenesis) (*50*). Both of these models have been implicated as mechanisms of alveolar neovascularization in compensatory lung regrowth (*51*, *52*). It is entirely possible that this process after viral injury is angiogenic only in the sense that nascent ECs are derived from preexisting endothelium as opposed to transdifferentiation of other cell types. Future studies using high-resolution, live imaging approaches will likely shed insight into the mode or spatiotemporal manifestation of this angiogenic process. Notably, proliferative ECs are mainly observed in areas of epithelial damage marked by loss of type 1 cells. ECs are certainly injured in these same regions, and proliferation of surviving ECs in these areas may also be promoted by factors derived from inflammatory cells present in these lesions. At earlier times after infection, we also observed areas exhibiting lower VECad expression adjacent to epithelial destruction, which may be due to the disruption of alveolar structure leading to local blood microcirculation obstruction or hypoxic vasoconstriction, thereby causing ischemia and additional injury to peripheral ECs near this area. However, these features are temporary and largely absent by day 27. Further study will be required to determine the nature of the transient vascular disruption in these regions.

COUP-TF2, an orphan nuclear receptor, is expressed in various cell types in multiple tissues, where it has been implicated in the regulation of many physiological processes and pathological states including Duchenne muscular dystrophy (*53*), development and differentiation in multiple organs (*54*), liver fibrosis (*55*), endocardial cushion hypoplasia (*56*), and venous endothelial fate adoption in development (*57*). Whether COUP-TF2 is involved in viral pneumonia or lung injury generally has not previously been investigated. Our data show that COUP-TF2 (Nr2f2) is down-regulated in lung ECs after influenza injury and slowly recovers concurrent with endothelial proliferation. These findings are consistent with studies in both developing coronary arteries (*19*) and the infrarenal aorta (*8*), where deletion of even a single copy of COUP-TF2 resulted in significant loss of EC proliferation (*19*). Notably, we find that deletion of COUP-TF2 in human lung microvascular ECs (iMVECs) (*29*) in vitro or in EC-specific COUP-TF2 conditional KO mice greatly impairs EC proliferation and, in the latter, results in exaggerated weight loss, strongly decreased capillary oxygen saturation, and increased mortality following influenza infection. Unchallenged mice did not exhibit any overt phenotype 2 months after COUP-TF2 deletion, likely due to the quiescent state of the pulmonary ECs under normal conditions (*58*). This indicates that a key function of COUP-TF2 in the endothelium is to facilitate mitosis, a role also suggested by recent work in the aorta where COUP-TF2 is up-regulated following mechanical injury in arterial ECs normally lacking COUP-TF2, which the authors suggest marks a “return to a more primitive stage that was perhaps more permissive for entry into the cell cycle” (*8*).

Excessive proinflammatory cytokine production and release, i.e., “cytokine storms,” can occur in severe influenza-induced pneumonia and COVID-19 and is recognized as a predictor of morbidity and mortality (*59*, *60*). The endothelium has been implicated as a central orchestrator of this phenomenon (*61*). Many influenza-induced cytokines, such as IL-1β and TNF-α (*25*, *26*, *62*), act both as potent activators of NF-κB and are themselves NF-κB target genes, indicating the potential for uncontrolled feed-forward inflammatory activation (*63*). Our results showed that IL-1β and TNF-α inhibit COUP-TF2 expression in vitro, similar to previous observations in other tissues (*28*). We demonstrate that NF-κB inhibitors can block this inhibitory effect and ChIP-qPCR revealed that NF-κB p65 directly binds the COUP-TF2 promoter region, indicating that cytokine suppression of COUP-TF2 is mediated by activation of NF-κB. In addition, NF-κB activating cytokines also promote up-regulation of microRNA-302a, which in turn down-regulates COUP-TF2, suggesting an additional mechanism that may contribute to this effect (*28*). NF-κB can also be activated by influenza virus structural proteins during infection (*64*). Our findings implicating a NF-κB/COUP-TF2 axis in influenza-induced vascular injury repair likely has important clinical significance. However, recent work indicates that alveolar epithelial regeneration is positively regulated by these same proinflammatory cytokines (*65*). Therefore, EC-specific inhibition of NF-κB signaling would likely be necessary to provide therapeutic benefit in patients with viral pneumonia, e.g., COVID-19 and influenza, by maintaining endothelial COUP-TF2 expression and enabling earlier, more effective endothelial repair. Accumulated evidence indicates that EC-specific inhibition of NF-κB is beneficial in many pathological settings, including protection from atherosclerosis (*66*), enhancing functional hematopoiesis (*67*), and attenuating hypertension-induced renal damage (*68*).

COUP-TF2 can function as either a transcriptional activator or repressor (depending on cofactors) through direct binding to target gene promoters in diverse cellular contexts (*36*). Our data demonstrate that Ccnd1 and Nrp1 are directly targeted by COUP-TF2. CCND1 is a critical cell cycle regulator and loss or repression of CCND1 in ECs leads to inhibition of proliferation (*37*), whereas, in our model, NRP1 has a larger role in EC migration. NRP1 was initially identified as a multifunctional receptor, expressed in neurons, blood vessels, immune cells, and many other cell types, which regulates organ development and function by binding structurally and functionally distinct ligands, including secreted semaphorins (Sema), VEGF, and VEGF family members including placenta growth factor PlGF (*69*). NRP1 is recognized as an important angiogenic factor, serving as a co-receptor for VEGFA_165_ (*39*), and VEGF-NRP1 signaling is required for angiogenesis and tip cell function in several ECs (*69*, *70*). Notably, NRP1 activation by Sema ligands does not affect blood vessel development but is instead essential for axon pathfinding of multiple neuron populations (*69*), suggesting that NRP1 downstream signaling is ligand dependent. While NRP1 signaling is required for physiological angiogenesis, abnormal or ectopic expression of NRP1 can also promote pathologic angiogenesis. For instance, in a mouse model of angioproliferative retinopathy, selective blockade of NRP1 substantially inhibited neovascular formation, limiting disease (*71*). In addition, NRP1 is up-regulated in clinical tumor samples (*72*). Recent studies also indicate that SARS-CoV-2 spike protein can also bind NRP1 in pulmonary vagal neurons, blocking nociceptive signaling and thereby potentially contributing to “asymptomatic” virus spread (*73*). Our data demonstrate that COUP-TF2 drives NRP1 expression, facilitating lung vascular repair, although the body of literature suggests that COUP-TF2–mediated NRP1 signaling may promote pathologic angiogenesis in other lung disease contexts, possibly pulmonary hypertension or lung tumor angiogenesis.

VEGFA is a potent mediator of both angiogenesis and vasculogenesis in the fetus and adult, with VEGFA_165_ the most abundant and potent isoform (*74*). Adenovirus-mediated VEGFA_165_ gene transfer enhances wound healing by promoting angiogenesis in vivo (*75*). It has been well demonstrated that VEGFA_165_ can enhance downstream angiogenesis events through NRP1/VEGFR2 signaling (*76*, *77*). Our results reveal that knockdown of COUP-TF2 leads to down-regulation of NRP1, attenuation of VEGFA_165_-mediated phosphorylation of VEGFR2, and ultimately inhibition of downstream proliferation and migration. Overexpression of COUP-TF2 did not significantly enhance VEGFA/NRP1/VEGFR2-mediated downstream angiogenic events. However, since COUP-TF2 also directly activates CCND1 to promote cell cycle entry, it is likely that the cell-autonomous increase in proliferation due to CCND1 up-regulation simply overwhelmed any additional effects mediated by NRP1 in vitro. The in vivo effects of COUP-TF2 during endothelial repair are thus likely mediated by both cell-autonomous (CCND1) and non–cell-autonomous (VEGFA/NRP1/VEGFR2) pathways.

Since COUP-TF2 is, in principle, ligand binding, identification of a specific COUP-TF2 ligand may very well serve as a potential therapeutic option for several viral pneumonia or ARDS. Although specific ligands for COUP-TF2 have not yet been identified, it has been shown that retinoids can promote COUP-TF2 to recruit coactivators and activate a COUP-TF2 reporter construct, although the concentration needed is higher than the physiological levels of retinoic acids (*78*). This begs the question as to whether Food and Drug Administration–approved retinoid molecules might be worth investigating and repurposing as an endothelial-focused treatment for severe lung injury.

Recent work has identified a specialized subset of capillary ECs in the murine lung marked by expression of Car4 (carbonic anhydrase 4). These cells appear to be specialized to enable high-efficiency gas exchange, in part due to their close apposition to alveolar type 1 cells without intervening pericytes and their extremely thin basement membrane (*18*). Pseudo-time analysis of single EC transcriptomes suggests that both these cells and traditional microvascular capillaries are capable of proliferation after influenza injury, and imaging indicated that Car4^+^ ECs are enriched in the most severely injured alveoli after infection (*79*). These cells are dependent on epithelial-derived VEGFA, and failure to specify these cells during development resulted in emphysema-like alveolar enlargement. It will be interesting to determine whether COUP-TF2 is required for the reestablishment of this population after viral injury, as might be suggested by their dependence on VEGFA.

There are several notable limitations to these studies that should be addressed in future work. While we expect these results to be generalizable to other etiologies of lung injury, it is possible that other pathogens or sterile injuries invoke different modes of endothelial repair, which will have to be determined on a per case basis. While native ECs are the sole source of endothelial repair after influenza, it is not certain whether every EC is equally “potent” to reenter the cell cycle after injury or whether specialized progenitor subsets may exist. Moreover, while we reproduce our murine in vivo results with human lung microvascular cells in vitro, it is, nonetheless, not currently possible to definitively demonstrate that these findings are completely recapitulated in human lung pathogenesis in vivo.

In summary, our studies establish the groundwork for a model of pulmonary vascular repair after viral pneumonia or ARDS. Our demonstration that angiogenic proliferation is the primary mode of vascular repair will allow future studies to focus on the endothelium itself as the source of capillary regeneration, although it leaves open the possibility that specialized progenitor cell subsets within the native endothelium are especially significant contributors. We further identify COUP-TF2 as a critical molecular target by which lung vascular repair might be enhanced to improve patient survival after viral lung injury. Hence, these studies should inform future approaches designed to promote endothelial regeneration and enhance EC potential to enable effective lung repair and prevent mortality in ARDS. As proper lung function requires the synergistic cooperation of epithelial, stromal, endothelial, and immune cells, the endothelium must be given due consideration in future efforts to enable and enhance lung regeneration after severe injury, viral, or otherwise.

## MATERIALS AND METHODS

### Study design

The objective of this study was to investigate lung vascular injury and underline repair mechanisms during viral pneumonia using an influenza virus–induced lung injury mouse model. For in vivo studies, experimental and control animals are specifically described as such in the Results and figure legends. Control mice for in vivo COUP-TF2 deletion experiments (COUP-TF2^EC−/−^) were an approximately equal mix of COUP-TF2^flox/flox^ mice lacking Cre and VECad^CreERT2^ mice bearing only WT COUP-TF2 alleles. Control animals were always treated identically including the same dosing of tamoxifen (see below). For in vitro studies, the experimental samples were COUP-TF2 KO or overexpression iMVECs, and iMVECs treated with indicated molecules or human lung primary ECs transfected with si-Nr2f2. Control samples were the corresponding empty vector–transduced iMVECs and/or vehicle-treated (dimethyl sulfoxide or PBS depending on the reagent) iMVECs or human lung primary ECs transfected with siRNA negative control (si-NC). Sample size was determined by availability and previous experience with influenza infection experiments in mice. No outliers were excluded from the study. A minimum of three animals per group was used for studies involving statistical analyses, and the *n* for individual experiments is indicated in the figure legends. Blinding was performed during data collection and analysis when possible, given the survival and body weight loss differences in treated and untreated groups. For each experiment, sample size reflects the number of independent biological replicates.

### Animals and treatment

COUP-TF2^flox^ mice (*24*) and lsl-Ai14-tdTomato(lsl-tdTomato) mice (*80*) were crossed with VECad^CreERT2^ (Cdh5^CreERT2^) mice (*81*). These crosses produced VECad^CreERT2;lsl-tdTomato^ mice and VECad^CreERT2^;COUP-TF2^flox/flox^ mice. VECad^CreERT2;lsl-tdTomato^ mice were administered three doses of body weight tamoxifen (0.25 mg/g) in 50 μl of corn oil every other day, followed by 3 weeks of “chase” time after the last injection. VECad^CreERT2^;COUP-TF2^flox/flox^ mice were administered five doses of body weight tamoxifen (0.25 mg/g) in 50 μl of corn oil every other day and rested for 2 to 8 weeks after the last injection, resulting in EC-specific deletion of *COUP-TF2* in adult mice (COUP-TF2^EC−/−^). Afterward, influenza virus A/H1N1/PR/8 was administered intranasally at 50 to 60 U of median tissue culture infectious dose to mice (20 to 25 g, 50 U; 25 to 30 g, 60 U) as our previously described (*50*, *80*). Control group mice were administered the same volume of PBS. Mice were weighed three times per week and euthanized at the indicated time points for tissue harvest. In this study, all mice were used at 6 to 8 weeks old, and mice of both sexes were used in equal proportions. All animal experiments were carried out under the guidelines set by the University of Pennsylvania’s Institutional Animal Care and Use Committees and followed all National Institutes of Health (NIH) Office of Laboratory Animal Welfare regulations.

### CRISPR-Cas9 editing in vitro

gRNAs targeting human COUP-TF2 for Cas9-mediated CRISPR disruption were designed using Benchling software. gRNA sequences are listed in table S1. The transduction of lentiviral gRNA + Cas9 followed a previous protocol and with appropriate modification (*83*). Briefly, gRNA oligos were synthesized, annealed, and ligated into LentiCRISPR V2-Puro plasmid (Addgene). Lentivirus was prepared by cotransfection of lentiviral plasmids with psPAX2 (Addgene) and pMD2.G (Addgene) packaging plasmids into 60 to 80% confluent 293FT cells using polyethylenimine as per the manufacturer’s protocol. Lentiviral supernatant was collected at 48 and 72 hours after transfection and used to transduce iMVECs in the presence of polybrene (10 μg/ml) (MilliporeSigma, #TR-1003-G). Stable COUP-TF2 KO cell lines (COUP-TF2-KO iMVECs) were selected by puromycin resistance (2 μg/ml) for 7 days and used for subsequent studies.

### Cell culture conditions

Primary human lung microvascular ECs were provided by S. H. Randell (Marsico Lung Institute, The University of North Carolina at Chapel Hill, USA). The cells were obtained under protocol no. 03-1396 approved by the University of North Carolina at Chapel Hill Biomedical Institutional Review Board as previously described (*84*). All donors or their authorized representatives provided informed consent for research use of explanted lungs. Briefly, human lung microvascular ECs were obtained by dispase and elastase digestion of peripheral lung tissue stripped of the visceral pleura, followed by primary culture in EGM-2 media with fetal bovine serum (Lonza). The cells were subjected to two or three rounds of CD31 bead purification (Dynabeads, Life Technologies/Invitrogen, #11155D), after which they were >95% CD31^+^ as measured by flow cytometry and used between passages 5 and 10. iMVECs were gifted by N. Mangalmurti. pLV-mNr2f2 (VectorBuilder, #VB190619-1154eaz) was used with the pMD2.G and psPAX2 plasmids to produce lentiviral particles for generation of the COUP-TF2-OE stable iMVECs cell line. Stable cell lines were selected by puromycin resistance (2 μg/ml) for 7 days. All cells were cultured in endothelial growth media (Lonza, #CC-3202). 293FT cells (Thermo Fisher Scientific) were cultured in Dulbecco’s modified Eagle’s medium (DMEM) (Thermo Fisher Scientific, #11965118) containing 10% cosmic calf serum (CC; HyClone, #SH3008704) and 1% penicillin/streptomycin (P/S; Gibco, #15140122). Depending on the experiment, iMVECs were treated with BAY-117082 (5 μM; Cayman Chemical, #10010266), JSH-23 (5 μM; Cayman Chemical, #15036), recombinant human TNF-α (100 ng/ml; ProSpec, #CYT-114), recombinant human IL-1β (100 ng/ml; ProSpec, #CYT-094), recombinant VEGFA_165_ (20 ng/ml; PeproTech, #100-20), ATWLPPR Peptide TFA (100 μM; MedChemExpress, #HY-P1663A), or vehicle control.

### Whole lung cell suspension preparation

Lungs were harvested from mice, and single-cell suspensions were prepared as previously described (*82*). Briefly, the lungs were thoroughly perfused with cold PBS via the left atrium to remove residual blood in the vasculature. Lung lobes were separated, collected, and digested with dispase II (15 U/ml) (Thermo Fisher Scientific, #17105041) in PBS for 45 min at room temperature and mechanically dissociated by pipetting in sort buffer (DMEM + 2% CC + 1% P/S; referred to as “SB”). Next, cell suspensions were filtered by the 40-μm cell strainer (Thermo Fisher Scientific, #352340) and treated by red blood cell lysis buffer (Thermo Fisher Scientific, A1049201) for 5 min, and the cell suspension was incubated in SB containing 1:1000 deoxyribonuclease I (DNase I) (MilliporeSigma, #D4527) for 45 min at 37°C. Whole lung cell suspensions were then used for subsequent experiments.

### Fluorescence-activated cell sorting

Whole lung single-cell suspensions were prepared as above and then blocked in SB containing 1:50 TruStain FcX (anti-mouse CD16/32) antibody (BioLegend, #101319) for 10 min at 37°C. The cell suspension was stained using allophycocyanin (APC)/Cy7–conjugated rat anti-mouse CD45 antibody (1:200; BioLegend, #101319), Alexa Fluor 488–conjugated rat anti-mouse CD31 [platelet endothelial cell adhesion molecule 1 (PECAM1)] antibody (1:200; BioLegend, MEC13.3, #102513) for 45 min at 4°C. Stained cells and “fluorescence minus one” controls were then resuspended in SB + 1:1000 DNase + 1:1000 Draq7 (BioLegend, #424001) as a live/dead stain. All FACS sorting was performed on a BD FACSAria Fusion Sorter (BD Biosciences).

### Intracellular FACS analysis

For intracellular EdU cytometry flow, *C57BL*/*6* mice were injected intraperitoneally with EdU (50 mg/kg; Santa Cruz Biotechnology, #sc-284628) at indicated time points. After euthanasia, the whole lung single-cell suspension was prepared as above and fixed by 3.2% paraformaldehyde (PFA) (Electron Microscopy Sciences, #15714-S) for 15 min, washed twice using 3% bovine serum albumin (BSA) (in PBS), and permeabilized using 0.1% Triton X-100 (in PBS) for 15 min. EdU was detected using the Click-iT reaction coupled to an Alexa Fluor 647 azide following the instructions of the manufacturer (Invitrogen, #C10086). For VECad-CreERT2 lineage analysis, lung cell suspensions were prepared as above. Cells were blocked in SB containing 1:50 TruStain FcX (anti-mouse CD16/32) antibody (BioLegend, #101319) for 10 min at 37°C. The cell suspension was stained using APC/Cy7-conjugated rat anti-mouse CD45 antibody (1:200; BioLegend, #101319), Alexa Fluor 488–conjugated rat anti-mouse CD31 (PECAM1) antibody (1:200; BioLegend, MEC13.3, #102513) for 45 min at 4°C. Intracellular flow analyses were performed on BD FACS Canto II flow cytometer (BD Biosciences).

### Immunofluorescence

For tissue sections, lungs were isolated and processed as previously described (*50*). Freshly dissected mouse lungs were fixed, embedded and cut into 7 or 50 μm (for confocal microscopy) thick cryosections, and postfixed another 5 min with 3.2% PFA. For immunostaining of in vitro experiments, cells were cultured on 24-well chamber slides. At the experimental end point, the cells were fixed with 3.2% PFA for 15 min. EdU incorporation was analyzed using the Click-iT reaction coupled to an Alexa Fluor azide following the instructions of the manufacturer (Invitrogen, #C10086) and followed by subsequent immunostaining. Both tissue sections and cell culture samples were blocked in PBS + 1% BSA (Gold Biotechnology, #A-420-100), 5% donkey serum (Sigma-Aldrich, #D9663), 0.1% Triton X-100, and 0.02% sodium azide for 1 hour at room temperature. Afterward, slides were probed with primary antibodies (CD45 1:200, BioLegend, #103107; CD31 1:200, BioLegend, #102513; VECad 1:200, R&D systems, #AF1002-SP; hSox17 1:400, R&D systems, #AF1924-SP; COUP-TF2 1:200, Abcam, #ab211777; Ki67 1:500, Thermo Fisher Scientific, #41-5698-82; ERG 1:2000, Abcam, #ab92513; RAGE 1:1000, R&D systems, #MAB1179) and incubated overnight at 4°C. The next day, slides were washed and incubated with the fluorophore-conjugated secondary antibodies (typically Alexa Fluor conjugates, Life Sciences) at a 1:1000 dilution for ≥1 hour. Last, slides were again washed, incubated with 1 μM 4′,6-diamidino-2-phenylindole (DAPI) for 5 min, and mounted using ProLong Gold (Life Sciences, #P36930). Images were taken with a microscope Dmi8 (Leica) and analyzed by LAS X software (Leica) and ImageJ (NIH) software.

### Histological analysis

Lung tissue sections fixed with 3.2% PFA were stained with hematoxylin and eosin by the Penn Vet Comparative Pathology core and then imaged with a Leica DMi8 microscope.

### BALF total protein quantification

The trachea was exposed, and a 20-gauge catheter was inserted for lavage. Cold PBS (1 ml) was instilled into the mouse lungs, and gentle aspiration was repeated three times. Total protein in BALF was determined by the bicinchoninic acid (BCA) colorimetric assay (*85*) using Pierce BCA Protein Assay Kit (Thermo Fisher Scientific, #23227).

### Pulse oximetry

Repeated measurements of peripheral oxygen saturation (SpO_2_) were taken using a MouseOx Plus rat & mouse pulse oximeter and a MouseOx small collar sensor (Starr Life Sciences Corp.). Mice were shaved around the neck and shoulders where the collar sensor sits. Recordings were taken using MouseOx Premium Software (Starr Life Sciences Corp., Oakmont, PA, USA). Measurements were taken continuously for >3 min at a measurement rate of 15 Hz. Measurements were imported into Microsoft Excel, and all readings with a nonzero error code were filtered out. The average of these error-free readings was used to calculate the SpO_2_ reading for each mouse for each given time point.

### siRNA transfection

RNA interference–mediated gene knockdown was performed using the validated si-Nr2f2 (Thermo Fisher Scientific, #4390824). After cells reached 60 to 80% confluence, the primary ECs at passage 5 were transfected with 50 pmol of the si-Nr2f2 or si-NC (Thermo Fisher Scientific, #4390843) for 48 to 72 hours using Lipofectamine RNAiMAX transfection reagent (Invitrogen, #13778030) according to the manufacturer’s protocol. The efficiency of knockdown was confirmed by qPCR and immunostaining.

### CCK-8

Cell proliferation assays were conducted using the CCK-8 (Dojindo, #CK04-05) following the manufacturer’s protocol. iMVECs were seeded 1 × 10^5^ per well onto a 96-well plate with indicated treatment for 0, 24, 48, and 72 hours; the cells were treated with 100 μl of DMEM/F-12 with CCK-8 reagent (1:10, v/v) and incubated for 2 hours. To estimate cell numbers, the absorbance of each well was measured at a wavelength of 450 nm using a microplate reader (Bio-Rad).

### Migration assay

Wound healing or migration assay was performed by seeding 6 × 10^4^ cells into the Culture-Insert 2 well according to the manufacturer’s recommendations (ibidi, #80209). After cells attached, the insert was removed, and low serum (<1%) medium was added. Images were taking immediately after insert removal (0 hours) and after 24 hours for the end point. Migration distance (in micrometers) was measured using LAS X software (Leica).

### Western blotting

Total protein from cells was extracted by lysis in radioimmunoprecipitation assay buffer (Sigma-Aldrich, #R0278) with protease inhibitor cocktail (Cell Signaling Technology, #5872). Protein concentrations were determined using a BCA protein assay kit (Thermo Fisher Scientific, #23227). Samples with equal amounts of protein were fractionated on SDS–polyacrylamide gels (Bio-Rad, #4561084), transferred to polyvinylidene difluoride (MilliporeSigma, #IPVH00005) membranes, and blocked in 5% skim milk (Cell Signaling Technology, #9999s) in TBST (0.1% Tween 20 in tris-buffered saline) for 1.5 hours at room temperature. The membranes were then incubated at 4°C overnight with primary antibodies [phospho-VEGFR2 (Tyr^1175^) 1:1000, Cell Signaling Technology, #3770; VEGFR2 1:1000, Cell Signaling Technology, #9698; COUP-TF2 1:1000, Abcam, #ab211777; Nrp1 1:1000, ABclonal, #A16697; Ccnd11:1000, ABclonal, #A19038; β-actin 1:50000, ABclonal #AC026]. After the membranes were washed with TBST, incubations with 1:4000 dilutions (v/v) of the secondary antibodies were conducted for 2 hours at room temperature. Protein expression was detected using the ChemiDoc XRS^+^ System (Bio-Rad). β-Actin was used as a loading control. Immunoblot bands were analyzed for optical density using ImageJ (NIH) software.

### ChIP assay

We followed a combined ChIP protocol based on the protocols for SimpleChIP Plus Enzymatic Chromatin IP kit (Cell Signaling Technology, #9005S) and ChIP-IT High Sensitivity kit (Active Motif, #53040). iMVECs were cross-linked using 1% formaldehyde; chromatin was isolated from 2 million cells according to the SimpleChIP protocol. We then switched to ChIP-IT High Sensitivity kit protocol from that point forward. Sheared chromatin (120 μg) was immunoprecipitated using 5 μg of ChIP-grade antibody [COUP-TF2 (*86*), R&D Systems, #PP-H7147-00; NF-κB p65, Cell Signaling Technology, #8242], which has already been experimentally validated for ChIP sequencing or an equal amount of immunoglobulin G (IgG) isotype control (mouse IgG, R&D Systems, #MAB003; rabbit IgG, Cell Signaling Technology, #3900). ChIP-DNA was purified and eluted in 150 μl of elution buffer. The *Ccnd1* and *Nrp1* promoter regions were predicted using the University of California, Santa Cruz genome browser. The binding sites in the promoter regions were predicted by JASPAR database. Primers were designed and listed in table S1.

### RNA isolation and qPCR

Total RNA was extracted by ReliaPrep RNA Cell Miniprep kit according to the manufacturer’s recommendation (Promega, #Z6011) and then reverse-transcribed into complementary DNA using the iScript Reverse Transcription Supermix (Bio-Rad, #1708841). qPCR was performed using a PowerUp SYBR Green Master Mix and standard protocols on an Applied Biosystems QuantStudio 6 Real-Time PCR System (Thermo Fisher Scientific). Glyceraldehyde-3-phosphate dehydrogenase was used to normalize RNA isolated from iMVECs and primary ECs; RPL19 was used to normalize RNA isolated from mouse samples. The 2^−ΔΔ*C*t^ comparative method was used to analyze expression levels. The primers used are listed in table S1.

### RNA-seq analysis

Total RNA was isolated and quality checked, DNA libraries were prepared, and sequencing was performed on an Illumina HiSeq platform by GENEWIZ Co. Ltd. Raw data (raw reads) in fastq.gz format were processed through a general pipeline (*87*) on Galaxy platform (*88*) (https://usegalaxy.org/). Briefly, raw reads (fastq.gz) were processed with FastqGroomer (.fastq), quality was checked by FastQC, and then the trimmed sequence reads were mapped to the reference genome [Genome Reference Consortium Human Build 38 patch release 13 (GRCh38.p13)] via STAR aligner. HTSeq was then used to quantify the number of reads mapped to each gene. Last, normalization and differential expression were carried out using the DESeq2. The list of differentially expression genes (DEGs) was used as the input for downstream analysis such as Gene Ontology (GO) terms and gene set enrichment analysis for known pathways network analysis to study the interactions between the DEGs.

### Statistics

All statistical calculations were performed using GraphPad Prism. *P* values were calculated from unpaired two-tailed *t* tests or analysis of variance (ANOVA) for multivariate comparisons. Variance was analyzed at the time of *t* test analysis. These data are not included in the manuscript but are available upon reasonable request.

## Supplementary Material

http://advances.sciencemag.org/cgi/content/full/6/48/eabc4493/DC1

Table S1

Adobe PDF - abc4493_SM.pdf

Regeneration of the pulmonary vascular endothelium after viral pneumonia requires COUP-TF2

## SUPPLEMENTARY MATERIALS

Supplementary material for this article is available at http://advances.sciencemag.org/cgi/content/full/6/48/eabc4493/DC1
