# Supplementary material for: Regeneration of the pulmonary vascular endothelium after viral pneumonia requires COUP-TF2
Source: Sci Adv. 2020 Nov 25;6(48):eabc4493. doi: 10.1126/sciadv.abc4493 (PMC7688336; doi:10.1126/sciadv.abc4493)
Supplement: http://advances.sciencemag.org/cgi/content/full/6/48/eabc4493/DC1 [file supp_6_48_eabc4493__index.html]

Science Advances | Science AdvancesAAASSearchScience AdvancesMenu

## Supplementary Materials

# Regeneration of the pulmonary vascular endothelium after viral pneumonia requires COUP-TF2

Gan Zhao, Aaron I. Weiner, Katherine M. Neupauer, Maria Fernanda de Mello Costa, Gargi Palashikar, Stephanie Adams-Tzivelekidis, Nilam S. Mangalmurti, Andrew E. Vaughan

Download Supplement

**This PDF file includes:**

- Figs. S1 to S9

**Other Supplementary Material for this manuscript includes the following:**

- Table S1

**Files in this Data Supplement:**

- Adobe PDF - abc4493\_SM.pdf
- abc4493\_Table\_S1.xlsx
