# Supplementary material for: Regeneration of the pulmonary vascular endothelium after viral pneumonia requires COUP-TF2
Source: Sci Adv. 2020 Nov 25;6(48):eabc4493. doi: 10.1126/sciadv.abc4493 (PMC7688336; doi:10.1126/sciadv.abc4493)
Supplement: http://advances.sciencemag.org/cgi/content/full/6/48/eabc4493/DC1 [file supp_6_48_eabc4493__1.pdf]

Science Advances
AAAS

advances.sciencemag.org/cgi/content/full/6/48/eabc4493/DC1

Supplementary Materials for

Regeneration of the pulmonary vascular endothelium after viral pneumonia requires COUP-TF2

Gan Zhao, Aaron I. Weiner, Katherine M. Neupauer, Maria Fernanda de Mello Costa, Gargi Palashikar, Stephanie Adams-Tzivelekidis, Nilam S. Mangalmurti, Andrew E. Vaughan*

*Corresponding author. Email: andrewva@vet.upenn.edu

Published 25 November 2020, *Sci. Adv.* **6**, eabc4493 (2020)
DOI: 10.1126/sciadv.abc4493

The PDF file includes:

Figs. S1 to S9

Other Supplementary Material for this manuscript includes the following:

(available at advances.sciencemag.org/cgi/content/full/6/48/eabc4493/DC1)

Table S1

Supplementary figures

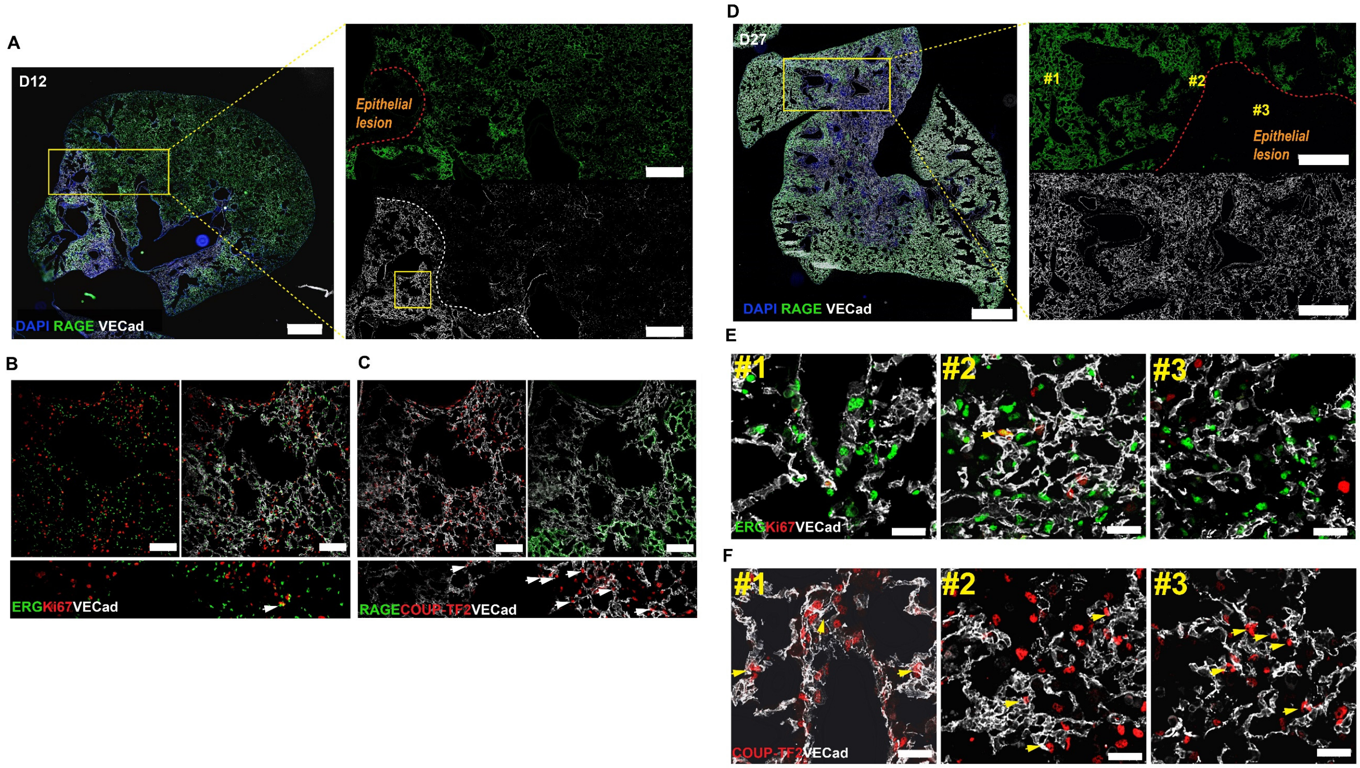

Fig. S1 Representative images indicating the distribution of proliferative ECs and COUP-TF2 staining in the lungs at 12 and 27 days after influenza infection. (A) Left: Influenza causes severe lung epithelial and endothelial injury on day 12 after influenza infection, scale bar: 1 mm; **Right:** Enlarged view of labeled part in left image. Scale bar: 500 µm. (**B**) Representative

immunostaining of proliferative ECs from epithelial lesion area on day 12 after influenza infection. Arrows indicate proliferative ECs (colocalization of ERG and Ki67), scale bar: 100 µm. (**C**) Immunofluorescence staining showing COUP-TF2 expression level in the capillaries in normal and injured epithelial regions on day 12 post-flu. Scale bar: 100 µm. Arrows indicate COUP-TF2 expression in capillaries. Bottom images in (B-C) are enlarged insets of figures above. (**D**) **Left:** lung endothelium (white: VECad) and epithelium (Green: RAGE) repair in mice recovered from influenza injury on day 27 post-infection, scale bar: 1 mm; **Right:** Enlarged view of labeled part in left image. Scale bar: 500 µm. (**E**) Representative immunostaining of proliferative ECs from healthy/recovered (#1) area or peripheral (#2) and central (#3) regions of epithelial lesion area on day 27 after influenza infection. Arrows indicate proliferative ECs (colocalization of ERG and Ki67), scale bar: 25 µm. (**F**) Representative immunostaining of COUP-TF2 in the capillaries from healthy/recovered (#1) part or peripheral (#2) and central (#3) parts of epithelial lesion area on day 27 after influenza infection. Scale bar: 25 µm. Arrows indicate COUP-TF2 staining in blood vasculature.

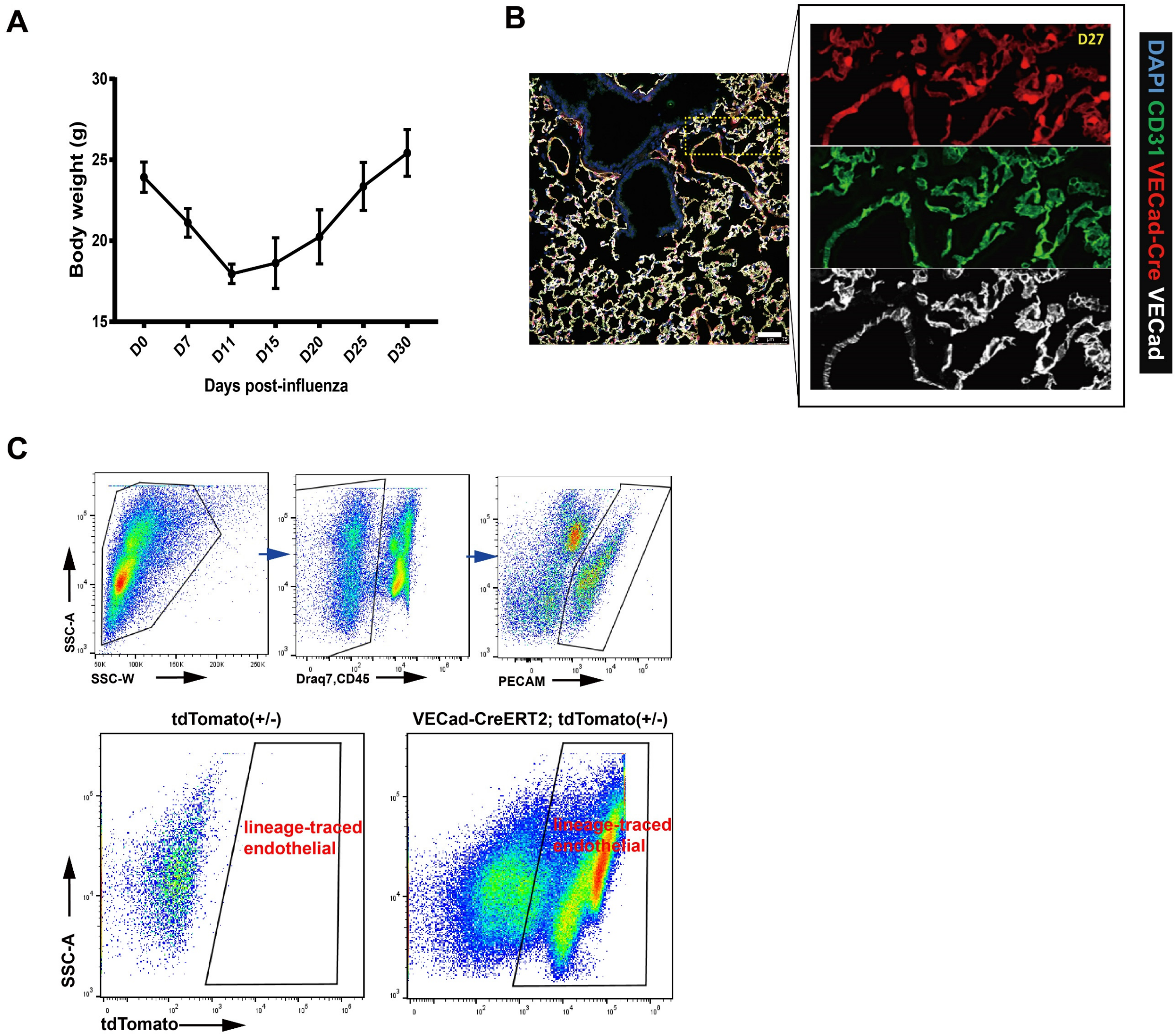

Fig. S2 Lineage trace analysis of regenerated ECs after influenza injury. (A) Time course change of bodyweight of VECad-CreERT2 lineage trace mice after influenza infection, n = 5. Data are presented as mean ± SD. (**B**) Immunostaining for endothelial-specific markers CD31 and VECad in VECad-CreERT2 lineage traced mice on day 30 after influenza injury, scale bar: 50 µm. Insets show magnified views of each channel. **(C)** Representative gating scheme for flow cytometry analysis of percentage of lineage traced ECs at baseline and after recovery from influenza.

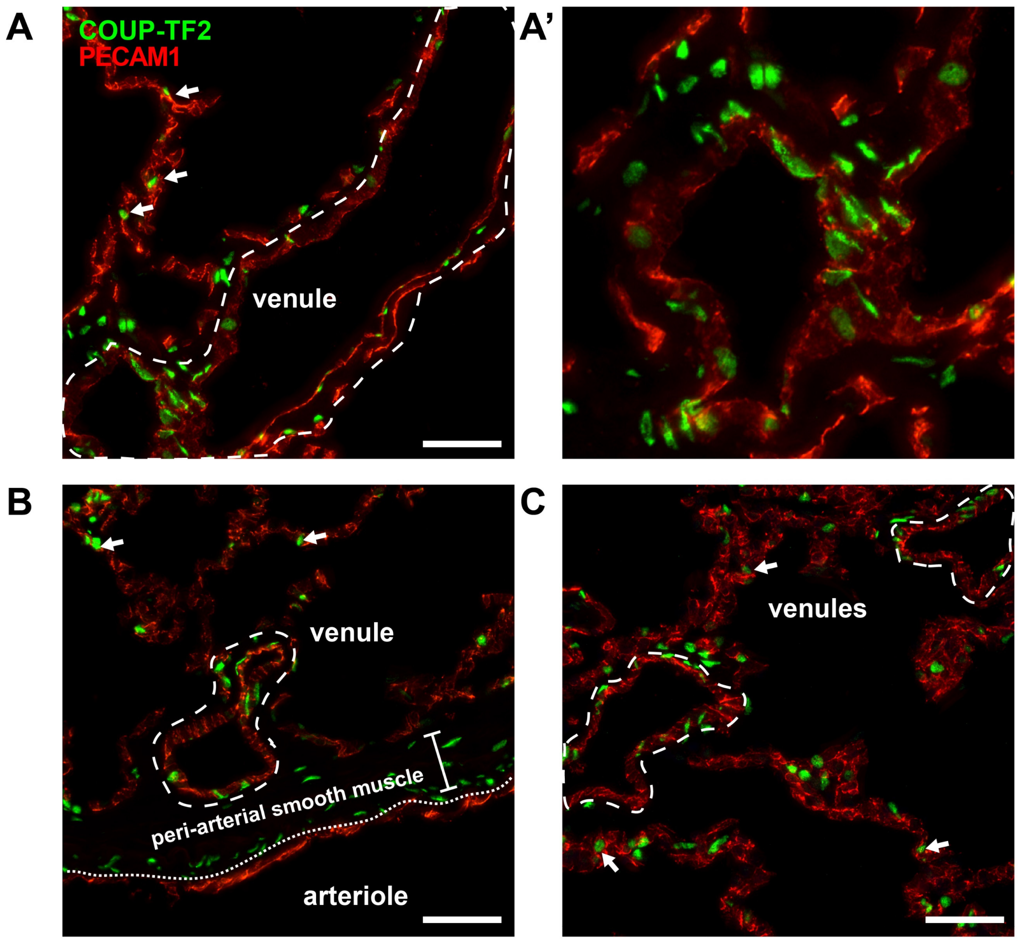

Fig. S3 COUP-TF2 is expressed in human pulmonary vascular endothelium. Human lung tissues were stained with COUP-TF2 and CD31/PECAM1, demonstrating COUP-TF2 expression in most lung endothelial cell types other than arteries (Capillary [white arrows], vein and lymphatic endothelial cells). **A, B, C** are 3 typical representative images. **A’** magnified views of **A**. Representative images from 2 distinct normal tissue donors, scale bar: 50 μm.

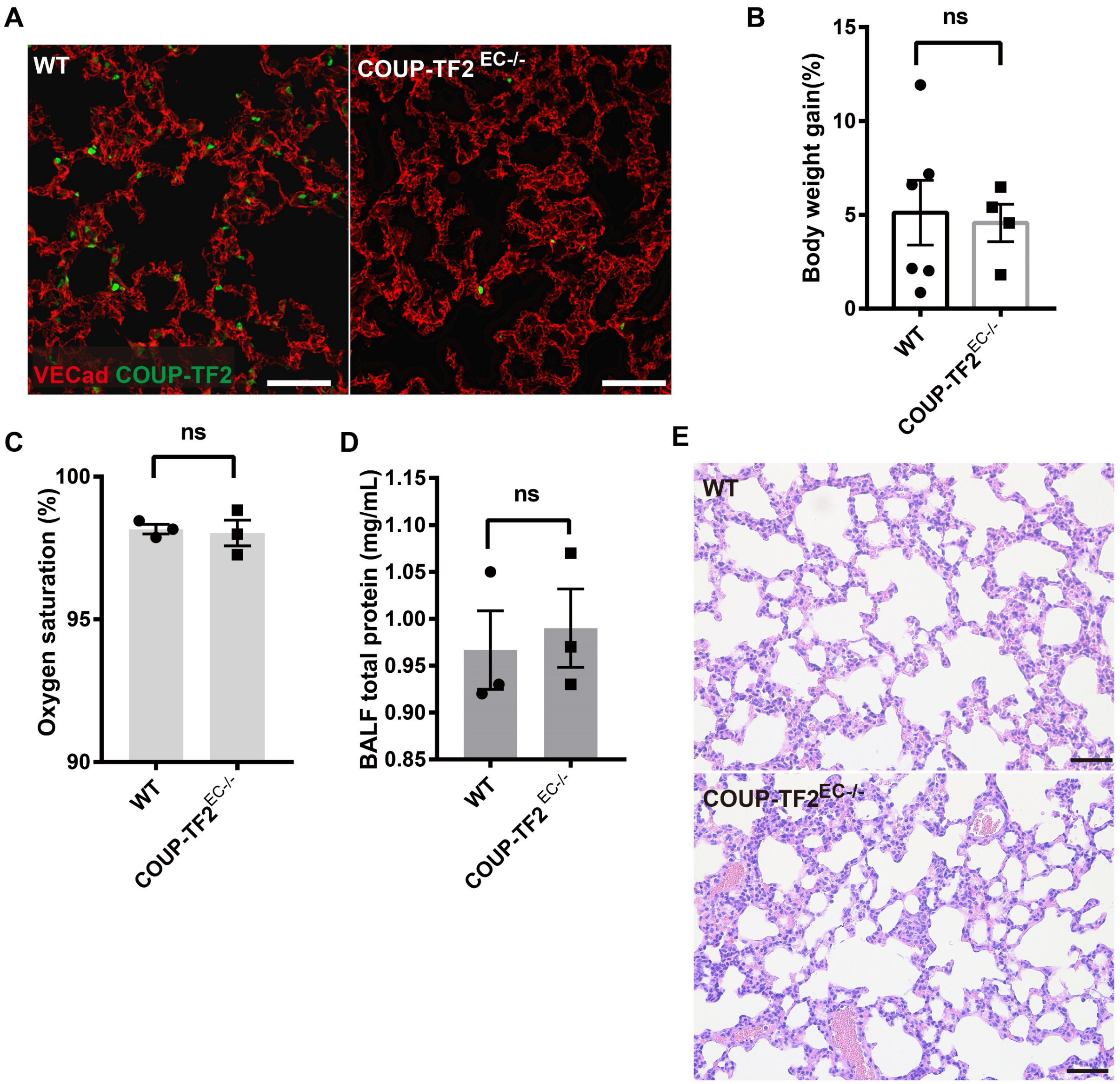

Fig. S4 Endothelial COUP-TF2 deletion without injury has little effect on pulmonary physiological function. (**A**) Lungs were harvested from WT and VECad^{CreERT2}; COUP-TF2$^{flox/flox}$ mice 4 weeks post-tamoxifen. Immunofluorescence imaging indicates that expression of COUP-TF2 in ECs was barely visible after tamoxifen administration in VECad^{CreERT2}; COUP-TF2$^{flox/flox}$ mice. Remaining COUP-TF2 positive cells represent mesenchymal or epithelial lineages already described to express COUP-TF2. (**B**) Mice were weighed on day 30 and 60 after the final dose of

tamoxifen. The body weight increase rate of COUP-TF2$^{EC-/-}$ mice is slightly lower than that of WT mice, no significant difference was observed (P > 0.05). Oxygen saturation (**C**) and total protein in lavage fluid (**D**) were quantified in WT and COUP-TF2$^{EC-/-}$ mice 4 weeks after the final tamoxifen administration and also demonstrate no difference. (E) H&E staining suggests that the alveolar structure and vascular network were clear and intact after COUP-TF2 deletion, but COUP-TF2$^{EC-/-}$ lungs may exhibit slight alveolar septal thickening, indicating the loss of COUP-4 TF2 in endothelial cells without challenge does not alter pulmonary physiology enough to cause obvious functional consequences, at least within this time period. Each point represents one mouse. Data are presented as mean ± SEM. *$P < 0.05$, **$P < 0.01$ calculating by unpaired two-tailed *t* test.

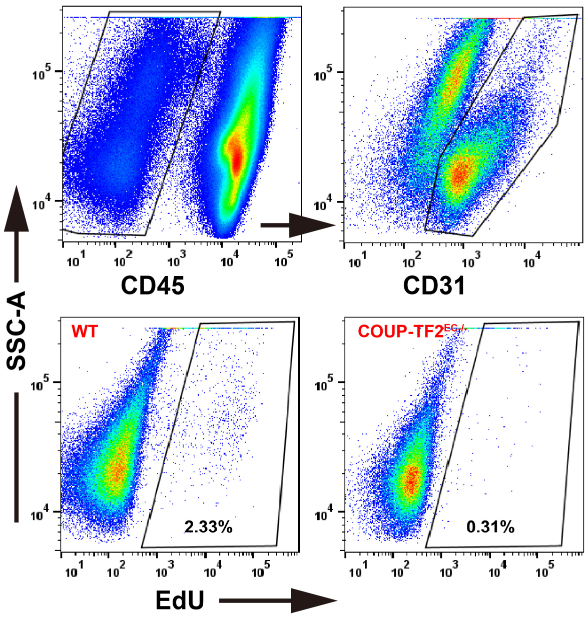

Fig. S5 Representative gating scheme for flow cytometry analysis of proliferative endothelial cells (CD45^{neg}/ CD31^{+}/EdU^{+}) in lungs from WT and COUP-TF2$^{EC-/-}$ mice.

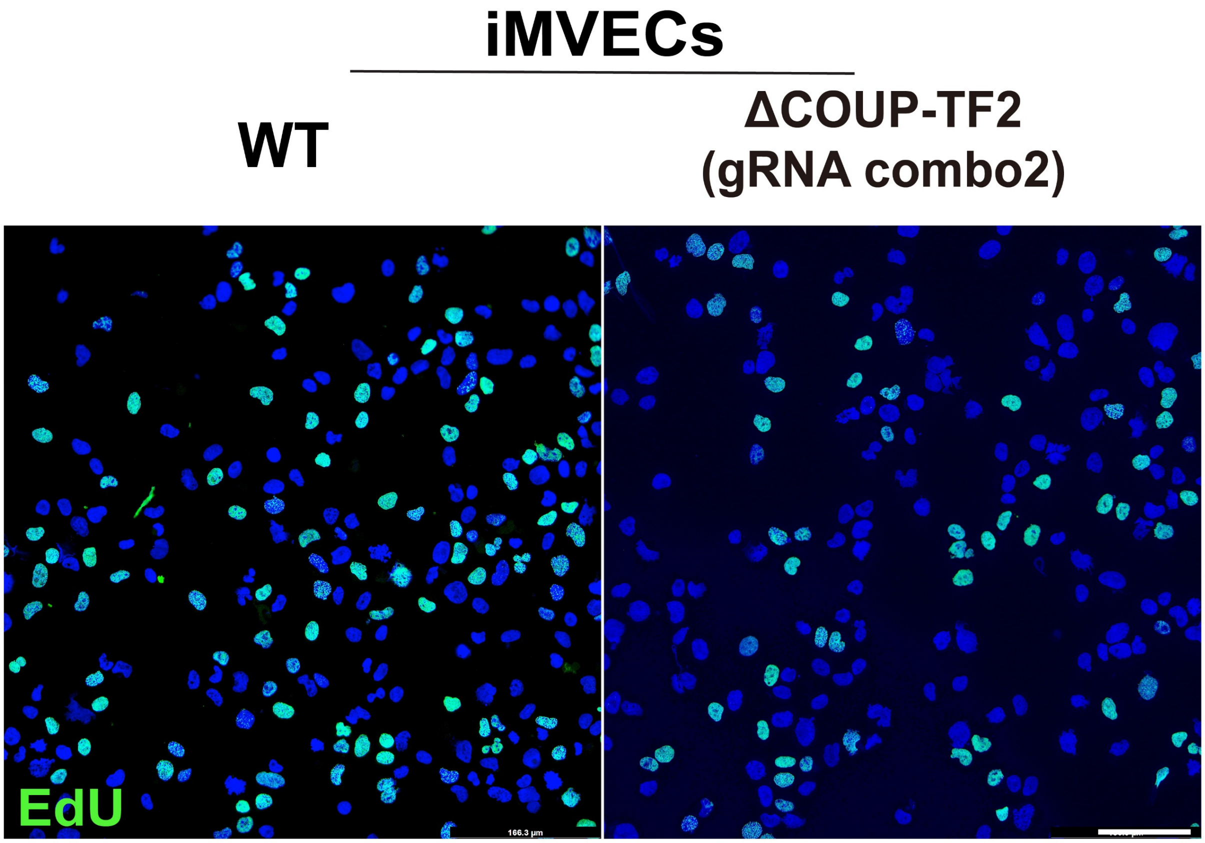

Fig. S6 Representative immunofluorescence for the nuclei (blue) and EdU incorporation (green) in WT and ΔCOUP-TF2 iMVECs.

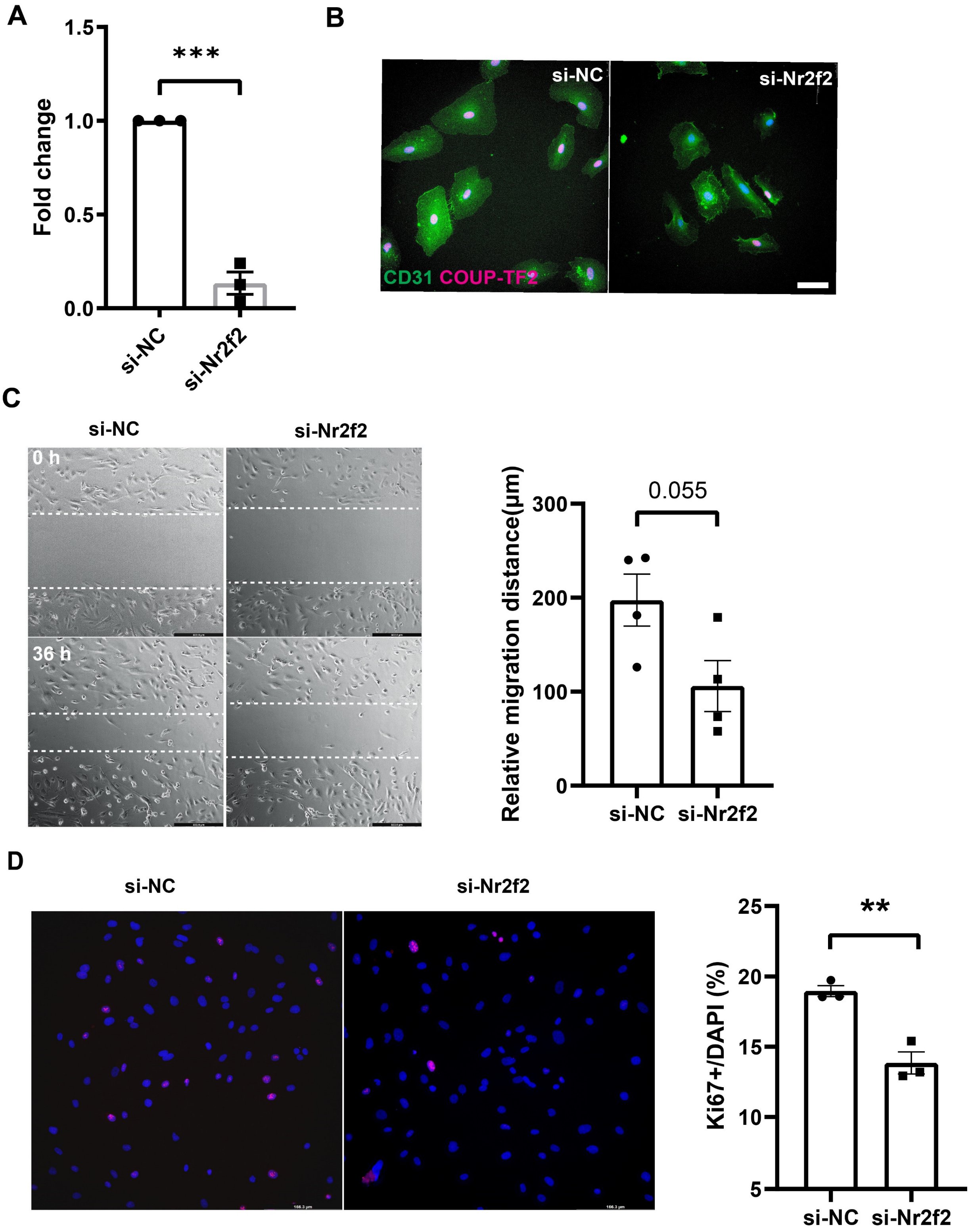
A
B
C
D
1.5
1.0
0.5
0.0
Fold change
***
si-NC
si-Nr2f2
CD31 COUP-TF2
0 h
36 h
Relative migration distance(μm)
0.055
300
200
100
0
Ki67+/DAPI (%)
**
25
20
15
10
5

Fig. S7 Knockdown of COUP-TF2 in human lung primary ECs inhibits proliferation and migration. (**A**) qPCR analysis of *COUP-TF2/Nr2f2* after transfection with si-Nr2f2 or si-NC. (**B**) Immunostaining for COUP-TF2 after transfection with si-Nr2f2 or si-NC. (**C**) Cell migration was assessed using a wound scratch assay. Images were obtained at (0 h) and (36 h). Representative photos illustrate scratch closing, quantified in the graph (right). (**D**) Quantification of proliferative ECs after COUP-TF2 knockdown through Ki67 immunostaining. Each dot represents one independent experiment with cells from one donor. Data are presented as mean ± SEM. $*P < 0.05$, $**P < 0.01$ calculating by unpaired two-tailed t test.

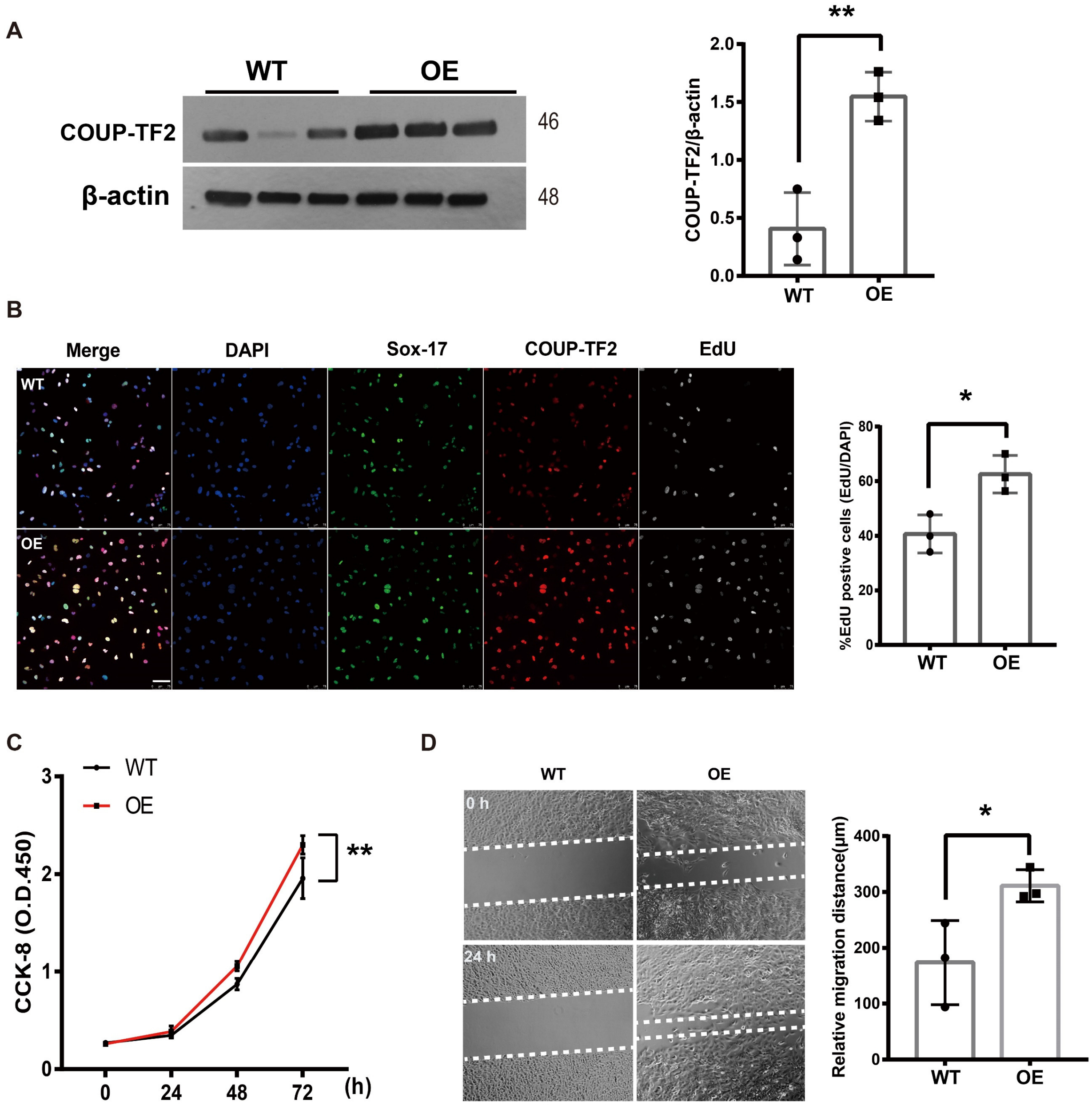

Fig. S8 COUP-TF2 overexpression promotes cell migration and proliferation. (**A**) COUP-TF2 was overexpressed in iMVECs by lentiviral transduction and confirmed by western blot (left); Quantification of COUP-TF2 protein by densitometry, normalized to actin (right). (**B**) Left: representative immunofluorescence for the nuclei (blue), Sox17 (green), COUP-TF2 (red) and EdU incorporation (white); Right: Quantification of proliferative endothelial cells (EdU/DAPI). (**C**) CCK-8 assay showing increased proliferation with COUP-TF2 overexpression.

(**D**) Cell migration was assessed by scratch assay. Images were taken at the start of the experiment (0 h) and 24 hours later (24 h). Photos of representative experiments are quantified in the graph (right), scale bar: 100 μm. Each dot represents one independent experiment. Data are presented as mean ± SD. *$P < 0.05$, **$P < 0.01$ calculating by unpaired two-tailed *t* test.

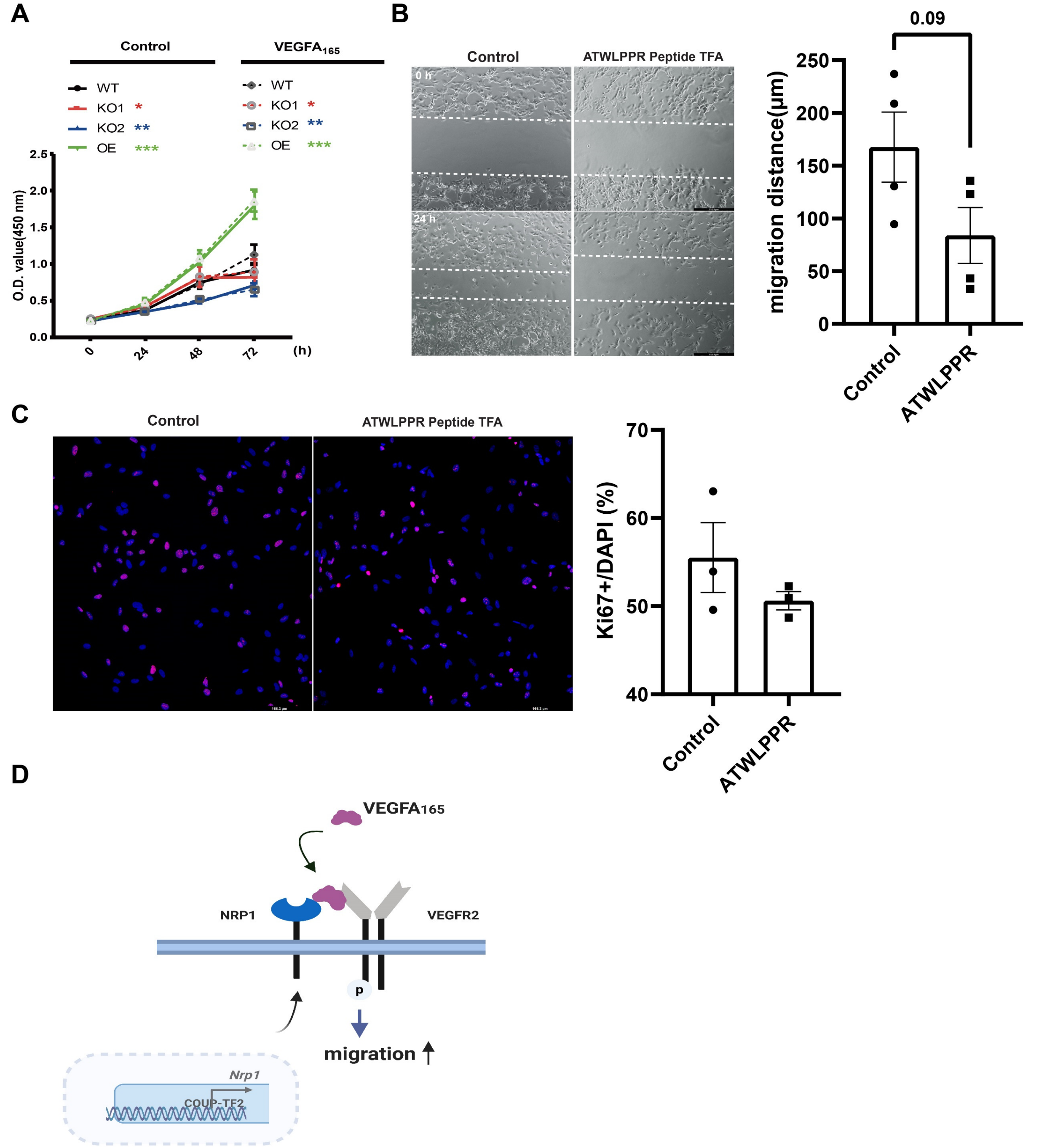

Fig. S9 NRP1 blocker partially inhibits $VEGFA_{165}$-induced cell migration. (A) $VEGFA_{165}$ promotes endothelial proliferation only with WT levels of COUP-TF2 expression. COUP-TF2-KO, OE and WT iMVECs were treated with $VEGFA_{165}$ (20 ng/mL) for 0, 24, 48, and 72 h, cell proliferation was assessed by CCK-8 assay. The proliferative effect of $VEGFA_{165}$ was only

apparent with endogenous levels of COUP-TF2 and was not observed in COUP-TF2 overexpression or knockout cells. (**B**) iMVECs were pre-treated with 100 μM NRP1 blocker ATWLPPR TFA and then incubated with $VEGFA_{165}$ (20 ng/mL), and cell migration was assessed using a wound scratch assay. Images were obtained at (0 h) and (24 h). Representative photos illustrate scratch closing, quantified in the graph (right). (**C**) Quantification of proliferative ECs through Ki67 immunostaining. (**D**) Proposed model of NRP1-mediated angiogenic migration and proliferation. Each dot represents one independent experiment. Data in A were calculated using two-way ANOVA followed by Dunnett's multiple comparison test. Data are presented as mean ± SD, n = 4. Data in **B** and **C** are presented as mean ± SEM. *$P < 0.05$, **$P < 0.01$ calculating by unpaired two-tailed t test. *$P < 0.05$, **$P < 0.01$, ***$P < 0.01$ vs. WT.
